# Integrated global analysis in spider flowers illuminates features underlying the evolution and maintenance of C_4_ photosynthesis

**DOI:** 10.1093/hr/uhad129

**Published:** 2023-06-20

**Authors:** Wei Zhao, Jun Li, Xingchao Sun, Qiwei Zheng, Jing Liu, Wei Hua, Jun Liu

**Affiliations:** Key Laboratory of Biology and Genetic Improvement of Oil Crops, Ministry of Agriculture and Rural Affairs, Oil Crops Research Institute of the Chinese Academy of Agricultural Sciences, Wuhan 430062, China; Key Laboratory of Biology and Genetic Improvement of Oil Crops, Ministry of Agriculture and Rural Affairs, Oil Crops Research Institute of the Chinese Academy of Agricultural Sciences, Wuhan 430062, China; Key Laboratory of Biology and Genetic Improvement of Oil Crops, Ministry of Agriculture and Rural Affairs, Oil Crops Research Institute of the Chinese Academy of Agricultural Sciences, Wuhan 430062, China; Key Laboratory of Biology and Genetic Improvement of Oil Crops, Ministry of Agriculture and Rural Affairs, Oil Crops Research Institute of the Chinese Academy of Agricultural Sciences, Wuhan 430062, China; Key Laboratory of Biology and Genetic Improvement of Oil Crops, Ministry of Agriculture and Rural Affairs, Oil Crops Research Institute of the Chinese Academy of Agricultural Sciences, Wuhan 430062, China; Hubei Hongshan Laboratory, Wuhan 430070, China; Key Laboratory of Biology and Genetic Improvement of Oil Crops, Ministry of Agriculture and Rural Affairs, Oil Crops Research Institute of the Chinese Academy of Agricultural Sciences, Wuhan 430062, China; Hubei Hongshan Laboratory, Wuhan 430070, China; Key Laboratory of Biology and Genetic Improvement of Oil Crops, Ministry of Agriculture and Rural Affairs, Oil Crops Research Institute of the Chinese Academy of Agricultural Sciences, Wuhan 430062, China

## Abstract

The carbon concentrating mechanism—C_4_ photosynthesis—represents a classic example of convergent evolution, but how this important trait originated and evolved remains largely enigmatic. The spider flower *Gynandropsis gynandra* is a valuable leafy vegetable crop and medicinal plant that has also been recognized as a C_4_ model species. Here we present a high-quality chromosome-scale annotated genome assembly of *G. gynandra* through a combination of Oxford Nanopore Technology (ONT), HiFi and Hi-C technology. The 17 super-scaffolds cover 98.66% of the estimated genome (997.61 Mb), with a contig N50 of 11.43 Mb and a scaffold N50 of 51.02 Mb. Repetitive elements occupy up to 71.91% of its genome, and over half are long terminal repeat retrotransposons (LTR-RTs) derived from recent bursts, contributing to genome size expansion. Strikingly, LTR-RT explosion also played a critical role in C_4_ evolution by altering expression features of photosynthesis-associated genes via preferential insertion in promoters. Integrated multiomics analyses of *G. gynandra* and the ornamental horticulture C_3_ relative *Tarenaya hassleriana* reveal that species-specific whole-genome duplication, gene family expansion, recent LTR–RT amplification, and more recent tandem duplication events have all facilitated the evolution of C_4_ photosynthesis, revealing uniqueness of C_4_ evolution in the *Cleome* genus. Moreover, high leaf vein density and heat stress resilience are associated with shifted gene expression patterns. The mode of C_3_-to-C_4_ transition found here yields new insights into evolutionary convergence of a complex plant trait. The availability of this reference-grade genomic resource makes *G. gynandra* an ideal model system facilitating efforts toward C_4_-aimed crop engineering.

## Introduction

Photosynthesis is the basis of most life forms on the planet. C_4_ photosynthesis represents a remarkable convergent innovation that results from a series of anatomical and biochemical modifications to the ancestral C_3_ photosynthetic pathway, which together function to increase CO_2_ concentration around the enzyme RuBisCO, thereby reducing photorespiration and enhancing photosynthetic efficiency [1]. Most, if not all, C_4_ species, which possess a distinctive leaf structure characterized by Kranz anatomy, are typically classified into three metabolic subtypes based on the enzymes used to decarboxylate C_4_ acids in their bundle sheath cells: NADP-ME, NAD-ME, and PEP-CK [2, 3]. C_4_ plants usually have higher photosynthetic capacity and higher nitrogen and water-use efficiencies than their C_3_ relatives and thus tend to be more productive [4, 5]. In particular, C_4_ photosynthesis outperforms the ancestral C_3_ state under sunny, hot, and dry circumstances, which are projected to become more prevalent with global climate changes [6, 7].

The introduction of dual-celled C_4_ photosynthesis into C_3_ plants is proposed to be a promising strategy to sustainably meet the rising food, fuel, and feed demands worldwide [8, 9]. This goal requires a profound understanding of the origin, genetic architecture, and developmental features of C_4_ syndrome as compared with C_3_. Several C_4_ monocots, including maize (*Zea mays*), sorghum (*Sorghum bicolor*), and millets (*Setaria viridis* and *Setaria italica*), have been used as model plants for this purpose [10–13]. However, these models share various disadvantages, including relatively large size, complex genomes, long life cycles, and low transformability [14]. Fortunately, a mini foxtail millet mutant, *xiaomi*, has recently been established as a new NADP-ME subtype C_4_ model system [15]. However, all current C_4_ model plants are monocotyledonous species, and there are differences in C_4_ attributes of dicots and monocots, including vein patterning, Kranz anatomy morphogenesis, regulation of C_4_ photosynthetic enzymes, and development of C_4_ capacity [1, 9]. Therefore, it is vital to develop a dicot C_4_ model plant and it would furthermore be advantageous to develop a model system in NAD-ME or PEP-CK subtypes of C_4_ plants. Investigation of these C_4_ model organisms would accelerate systematic understanding of C_4_ biology and facilitate synthetic engineering of the C_4_ pathway into contemporary C_3_ crops.

The dicot *Gynandropsis gynandra* (common names: spider flower, African cabbage, bastard mustard, and cat’s whiskers) is an important traditional C_4_ crop native to Asia and Africa, currently cultivated around the world [16]. The leaves and seeds of *G. gynandra* are rich in proteins, vitamins, minerals, and other beneficial health compounds with antioxidant, anti-inflammatory, and antimicrobial properties, and *G. gynandra* has thus been widely used as a leafy vegetable or medicinal plant [17]. As a C_4_ species, *G. gynandra* displays high photosynthetic efficiency and adaptation to severe environmental stresses such as high temperature, water deficit, or high salinity [18]. Importantly*,* it is a diploid crop characterized by a relatively short life cycle, small size, simple growth requirements, prolific seed production, self-compatibility, and autogamy. Besides, *G. gynandra* has a rich, diverse germplasm that provides valuable genetic material for dissecting traits of interest and breeding [19, 20]. These advantages, coupled with its efficient genetic transformation system, make this plant an ideal model plant for C_4_ biology [21].

*Gynandropsis gynandra* belongs to the family Cleomaceae, a sister clade to Brassicaceae, and is phylogenetically closest to the dicot C_3_ model plant *Arabidopsis thaliana*, enabling the utilization of molecular resources and tools developed for *Arabidopsis* in *G. gynandra* and facilitating knowledge transfer [22–24]. Moreover, *G. gynandra*, together with the ornamental horticulture C_3_ species *Tarenaya hassleriana* (common names: spider flower, pinkqueen, and grandfather's whiskers), provides an invaluable genetic platform for comparative studies of many intriguing biological phenomena, including the evolutionary trajectory and developmental progression from C_3_ to C_4_ [25–28]. However, there is no telomere-to-telomere gap-free genome available for *G. gynandra*, which hinders its use for fundamental biological discovery, technological advances, and applications for genetic engineering. To address this gap, we generated a high-quality chromosome-level reference genome assembly of *G. gynandra*, establishing it as a C_4_ model system for dicotyledonous and NAD-ME subtype plants. Our comprehensive comparative genomic and transcriptomic analyses between *G. gynandra* and *T. hassleriana* using new and existing data not only help explain why C_4_ photosynthesis failed to evolve in *T. hassleriana* but also demonstrate the flexibility of the convergent evolution of this ecologically important but complex trait.

## Results

### Chromosome-scale assembly and annotation of *G. gynandra* genome

**Figure 1:**
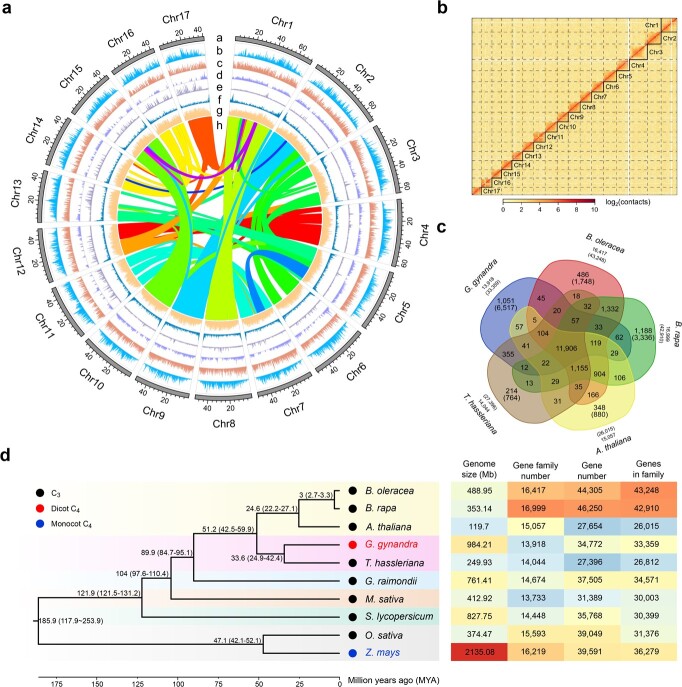
High-quality assembly, genome features, and evolutionary analysis of *G. gynandra*. **a** Overview of genomic characteristics of *G. gynandra*. The outermost annotations number successively the 17 assembled chromosomes in descending order of size. Tracks from outer to inner circles depict length (Mb) of each chromosome (a), *Gypsy* retrotransposon distribution (b), *Copia* retrotransposon distribution (c), DNA transposon distribution (d), tandem repeats (e), gene density (f), GC content (g), and curve lines in the interior linking the syntenic regions that have been retained presumably since the last whole-genome duplication event (h). Sliding window size is 200 kb. **b** Genome-wide Hi-C heat map of the *G. gynandra* genome. The heat map shows the diagonal pattern for strong Hi-C intra­chromosome interactions of *G. gynandra*. The *X*-axis and *Y*-axis indicate the order positions of scaffolds on corresponding pseudochromosomes. The color bar denotes the interaction frequencies of the Hi-C links. **c** Venn diagram illustrating the shared orthologous groups (orthogroups) among five species: *G. gynandra*, *T. hassleriana*, *B. rapa*, *B. oleracea*, and *A. thaliana*. Each number represents the number of gene families shared among genomes. The number listed in parentheses is the total gene number among the orthogroups. **d** Phylogenetic relationships of the two Cleomaceae and eight other species used in the study (*B. oleracea*, *B. rapa*, *A. thaliana*, *G. raimondii*, *M. sativa*, *S. lycopersicum*, *O. sativa*, and *Z. mays*). The phylogenetic tree was constructed from single-copy orthologs of these species. Lineage divergence time is indicated at each branch point. The photosynthesis subtype of each species is marked by colored dots at each node. The size, gene family number, gene number, and number of genes belonging to gene families for each species are listed in the heat map at right.

Based on *k*-mer analysis, the dicotyledonous C_4_ plant *G. gynandra* (2*n* = 2*x* = 34) had an estimated genome size of ~997.61 Mb with low heterozygosity (0.13%) but high repetitive sequence content (79.72%) (Supplementary Data Fig. S1, Supplementary Data Table S1). To construct a reference-grade genome for *G. gynandra*, we employed an optimized strategy combining long-read Oxford Nanopore Technology (ONT), short-read Illumina sequencing, high-throughput chromatin conformation capture (Hi-C) for chromosome scaffolding, and PacBio HiFi sequencing technology (Supplementary Data Fig. S2). A total of 144.37 Gb (~180×) of ONT sequences with an N50 read length of 24.57 kb were generated (Supplementary Data Fig. S3, Supplementary Data Table S2). The ONT long reads were *de novo* assembled into contigs, followed by polishing with both ONT and Illumina reads. To anchor and orient the contigs onto chromosomes, we prepared Hi-C libraries to construct chromatin interaction maps, generating 130 Gb (~162×) paired-end reads. We then performed HiFi sequencing to obtain a gapless *G. gynandra* genome. A total of 608.61 Gb (~618×) of HiFi reads with an N50 read length of 15.99 kb were generated (Supplementary Data Table S3). These resulted in a final assembly of 984.21 Mb comprising 109 scaffolds, which represented 98.66% of the estimated genome (Table 1, Supplementary Data Table S4). The contig N50 and scaffold N50 were 11.43 and 51.02 Mb, respectively. Sequences of 909.61 Mb covering 171 contigs were assigned to 17 pseudochromosomes (Chr1–Chr17; Fig. 1a, Table 1), which accounted for 92.42% of the assembly. The pseudochromosomes of *G. gynandra* ranged from 41.13 to 72.98 Mb in size. One putative centromere was identified for each of the 17 pseudochromosomes, with lengths of the centromeres ranging from around 2 to 4 Mb. Of note, two telomeres were identified at both ends of almost all the *G. gynandra* chromosomes (except for Chr14) (Supplementary Data Table S5). The entire genome assembly had only 11 gaps distributed on seven chromosomes, including 4 gaps within the extremely repetitive regions on Chr1. Collectively, these data indicate that the assembled *G. gynandra* genome can be considered a high-quality telomere-to-telomere genome.

**Table 1:** Global statistics of *G. gynandra* genome assembly and annotation.

**Genomic feature**	**Value**
Genome assembly	
Estimated genome size	997.61 Mb
Assembled genome size (≥1000 bp)	984.21 Mb
Assembled genome percentage	98.66%
GC content	39.24%
Total number of scaffolds	109
Scaffold N50	51.02 Mb
Longest scaffold	72.99 Mb
Total number of contigs	687
Contig N50	11.43 Mb
Longest contig	55.87 Mb
Sequence assigned to pseudochromosomes	909.61 Mb
Number of pseudochromosomes	17
Number of anchored and oriented contigs	171
Genome annotation	
Repetitive sequences	707.76 Mb
Repetitive sequences percentage	71.91%
Number of genes	34 772
Size of total gene length	119.12 Mb
Number of transcripts	41 843
Average length of transcripts	1824 bp
Non-coding RNAs	12 441

The assembly quality of the *G. gynandra* genome was evaluated by multiple approaches. First, Hi-C interaction matrices for the constructed pseudochromosomes visualized as a Hi-C heat map showed a clear anti-diagonal pattern for intra-chromosomal interactions (Fig. 1b). Second, the completeness of assembly as analyzed with Benchmarking Universal Single Copy Orthologs (BUSCO) was 95.6% (Supplementary Data Table S6). Third, RNA-seq reads obtained from six representative tissues of flower bud, flower, leaf, root, stem, and silique were mapped back onto the *G. gynandra* genome, to which ~96.16–98.33% of the reads could be aligned (Supplementary Data Table S7). Finally, the long terminal repeat (LTR) assembly index (LAI), a metric using intact LTRs to evaluate assembly continuity, has been particularly utilized to assess assembly quality of plant genomes with high repetitive sequence content [29]. The LAI score of the *G. gynandra* assembly was 16.46, higher even than that of the *A. thaliana* reference genome (LAI = 15.62). Collectively, these data demonstrate that the *G. gynandra* genome assembly is of high quality in contiguity, completeness, and accuracy.

By integrating *ab initio*-based prediction, protein homology-based prediction, and RNA-seq/Iso-Seq data, we annotated a total of 34 772 protein-coding genes spanning regions totaling 119.12 Mb, representing 12.1% of the *G. gynandra* genome (Table 1). The gene density distribution along each chromosome was uneven, with higher gene density towards the ends of chromosome arms (Fig. 1a). The total number of identified transcripts (including splicing variants) was 41 843. Functional analysis predicted 33 100 (95.19%) genes with known functional annotations in public databases, suggesting highly reliable gene prediction (Supplementary Data Table S8). Moreover, we identified 2359 non-coding RNAs, including 120 miRNAs, 1153 tRNAs, and 1086 rRNAs (Supplementary Data Table S9), and also 2178 transcription factors (Supplementary Data Table S10). Of all the protein-coding genes, 95.9% were assigned to 13 918 gene families in *G. gynandra*, which was comparable to *T. hassleriana* (97.8% to 14 044 gene families). However, the former had an average of 2.39 genes per family, with the latter possessing 1.91 (Supplementary Data Table S11). Compared with *A. thaliana*, *Brassica rapa*, *Brassica oleracea* and *T. hassleriana,* 1051 gene families containing 6517 genes were specific to *G. gynandra* (Fig. 1c). These unique genes were primarily enriched in metabolic pathways such as biosynthesis of amino acids, carbohydrates, lipids, cofactors and vitamins, terpenoids, and polyketides, which is consistent with the nutraceutical food and ethnopharmacological medicinal uses for *G. gynandra* (Supplementary Data Fig. S4).

Phylogenetic analysis was performed based on 661 single-copy orthologous genes among 10 angiosperm species, including eight dicots (*A. thaliana*, *B. oleracea*, *B. rapa*, *G. gynandra*, *T. hassleriana*, *Gossypium raimondii*, *Medicago sativa* and *Solanum lycopersicum*) and two monocots (*Oryza sativa* and *Z. mays*). The results revealed that *G. gynandra* was the most closely related to *T. hassleriana*, both of which were adjacent to *A. thaliana* (Fig. 1d). Using as a reference divergence times of *B. oleracea*–*B. rapa*, *Z. mays*–*O. sativa*, and *A. thaliana*–*O. sativa* obtained from the TimeTree database, the divergence between Cleomaceae and Brassicaceae was estimated as 51.2 million years ago (MYA), with C_4_*G. gynandra* and C_3_*T. hassleriana* sharing a common ancestor ~33.6 MYA. Remarkably, the genome size of *G. gynandra* (984.21 Mb) is nearly 4-fold as large as *T. hassleriana* (249.93 Mb).

### Recent bursts of transposons massively bloated the *G. gynandra* genome, and LTR-RTs facilitated the evolution of C_4_ photosynthesis

**Figure 2:**
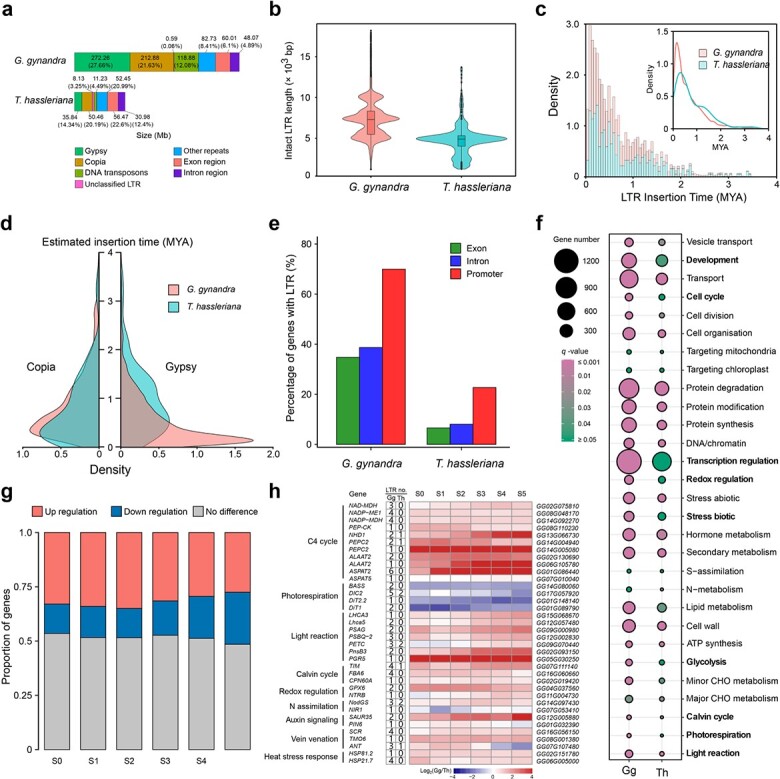
Comparative analysis of TEs in *G. gynandra* and *T. hassleriana* genomes, and the role of LTR-RTs in C_4_ photosynthesis evolution. **a** Genomic makeup by category of *G. gynandra* and *T. hassleriana.* The colored blocks indicate the sizes (Mb) of different components, including LTR-RTs (*Gypsy* and *Copia*), DNA transposons, unclassified LTR-RTs, other repeats, and coding regions. The number in parentheses is the percentage of the genome each constitutes. Each number denotes the size (Mb) of each composition*. Gypsy*, *Copia*, and DNA transposons were much more abundant in the *G. gynandra* genome than in *T. hassleriana*. **b** Length distribution of intact LTR-RTs in *G. gynandra* and *T. hassleriana.* Boxes within violin plots indicate the first quartile, the median, and the third quartile with whiskers extending up to 1.5× interquartile range (IQR). Outliers are shown as dots, defined as data points outside 1.5× IQR. The *G. gynandra* genome contained many more intact LTR-RTs >10 kb in length than *T. hassleriana*. **c** Estimated insertion times of LTR-RTs into *G. gynandra* and *T. hassleriana* genomes. The *X*- and *Y*-axes indicate the insertion times and the density of intact LTR-RTs at each time, respectively. *G. gynandra* underwent more extensive LTR-RT explosion than *T. hassleriana* during the last 2 million years. **d** Temporal patterns of *Gypsy* and *Copia* bursts in *G. gynandra* as compared with *T. hassleriana*. The *X*- and *Y*-axes indicate the density of *Gypsy*/*Copia* and the insertion times, respectively. Despite *G. gynandra* and *T. hassleriana* sharing similar density distributions of *Copia*, the former experienced a surge of *Gypsy* insertion very recently. **e** Percentage of LTR-RT exon-, intron-, or promoter-inserted genes. The 2-kb region upstream of the transcription start site is defined as the promoter region of the gene. Genes with LTR-RT insertion in promoter regions are much more abundant than in exons or introns, especially in *G. gynandra*. **f** MapMan-Bin enrichment of the LTR-RT promoter-inserted genes in *G. gynandra* and *T. hassleriana*. Annotations of protein sequences with MapMan terms were performed with the online Mercator (https://www.plabipd.de/portal/mercator4). The MapMan4 program was used to conduct the analysis, and the results were visualized with R software. The MapMan terms with clear differences between the two species are highlighted in bold. **g** Histograms showing the proportion of selected LTR-RT promoter-inserted genes with up- or downregulation in *G. gynandra* relative to *T. hassleriana*. The 6283 genes, which have more LTR-RT insertions in their promoter regions in *G. gynandra* than in *T. hassleriana*, were used for expression analysis*.* This was performed with the previously deposited RNA-seq data of leaves at six developmental stages (from young to mature, S0 to S5) for these two *Cleome* species [26]. The gene expression levels were normalized with the upper quartile normalization procedure using the youngest S0 leaf stage of *G. gynandra* as the reference. No difference is defined if the level of gene expression shows <1.5-fold change between *G. gynandra* and *T. hassleriana*. The *X*- and *Y*-axes indicate leaf developmental stages and the proportion of genes in each regulation category, respectively. The proportions of upregulated genes are overall higher than those of downregulated genes at all analyzed leaf stages. **h** Expression patterns of C_4_ photosynthesis-related pathway genes that have more LTR-RT insertions in their promoter regions in *G. gynandra* than in *T. hassleriana*. Pathways and member genes are indicated at left. The two columns of numbers show how many LTR-RTs were found in the promoter region of each gene in *G. gynandra* and *T. hassleriana*, respectively. The right panels show differential expression ratios of the genes between the two species at various leaf developmental stages (S0 to S5). Color reflects fold differences (log_2_ ratios) in gene expression. LTR no., number of LTR-RTs inserted.

By combining *de novo*- and homology-based repeat family identification approaches, we annotated a total of 707.8 Mb repetitive sequences, representing 71.91% of the *G. gynandra* genome (Table 1). We classified 613.5 Mb of sequences (62.33% of the total assembly) as transposable elements (TEs), including 485.73 Mb (79.17% of TEs) of LTR retrotransposons (LTR-RTs) and 118.88 Mb of DNA transposons, whereas their size and proportion were dramatically lower in *T. hassleriana* (Fig. 2a, Supplementary Data Table S12). The spatial distribution of LTR-RTs along chromosomes of *G. gynandra* was uneven (Supplementary Data Fig. S5). Its genome was found to contain a considerable number of intact LTR-RTs with sequence length up to 10 000 bp, with a peak at around 7500 bp, while the peak in *T. hassleriana* occurred at ~5000 bp (Fig. 2b). Most (79.72%) intact LTR-RT insertion events in the *G. gynandra* genome occurred within 1 MYA, with the peak of amplification occurring around 0.16 MYA, in contrast to ~0.31 MYA for *T. hassleriana* (Fig. 2c). At the superfamily level, very recent amplifications of *Gypsy* retrotransposons occurred ~0.14 MYA in *G. gynandra* (Fig. 2d). *Gypsy* retrotransposons had the highest distribution density in the centromeric area and the lowest toward telomeric regions, with *Copia* showing a continuous distribution pattern (Fig. 1a).

Recent massive expansion of LTR-RTs led us to explore their biological relevance. There were 20 094 homologous gene pairs in total between the genomes of *G. gynandra* and *T. hassleriana*. Relative to homologues without LTR-RTs, their presence caused more to be upregulated (Supplementary Data Fig. S6a). It should be noted that although *G. gynandra* has more exons and introns than *T. Hassleriana* (Fig. 2a), LTR-RTs were found to reside preferentially within regions of promoters relative to those of exons and introns, especially in *G. gynandra* (Supplementary Data Fig. S6b), potentiating their differential roles in regulating gene expressions between the two *Cleome* species. More than 70% of *G. gynandra* genes had LTR-RT insertions in promoter regions of protein-coding genes, which were only 23% for *T. hassleriana* (Fig. 2e). Functional enrichment analysis of these preferentially inserted genes indicated that pathways of development, cell cycle, transcription regulation, redox regulation, stress-related, glycolysis, Calvin cycle, photorespiration, and light reaction were over-represented in *G. gynandra* compared with *T. hassleriana* (Fig. 2f). Furthermore, we identified 6283 genes in *G. gynandra* whose promoter regions contained substantially more LTR-RT insertions than their orthologs in *T. hassleriana* (Supplementary Data Table S13). To assess the consequence of LTR-RT amplification in these genes, their expression levels were analyzed in *G. gynandra* relative to *T. hassleriana*. Moreover, we found that the proportions of genes with upregulated expression were much greater than those with downregulated expression across the six developmental stages, being more pronounced at the early stages (Fig. 2g). Notably, the significantly upregulated genes along the development gradients included a subset of genes associated with C_4_ metabolism (Fig. 2h and Supplementary Data Table S22). In contrast, photorespiration genes with more LTR-RTs were severely downregulated in *G. gynandra*. Additionally, LTR-RT insertions caused few C_4_ metabolism-related genes to be downregulated (Supplementary Data Fig. S6c). Together, these data reveal that recent large-scale TE bursts are the driving force behind genome size expansion and that LTR-RTs also play a role in the C_4_ evolution of *G. gynandra*.

### Evidence for species-specific whole-genome duplication and tandem gene duplication events in *G. gynandra*

**Figure 3:**
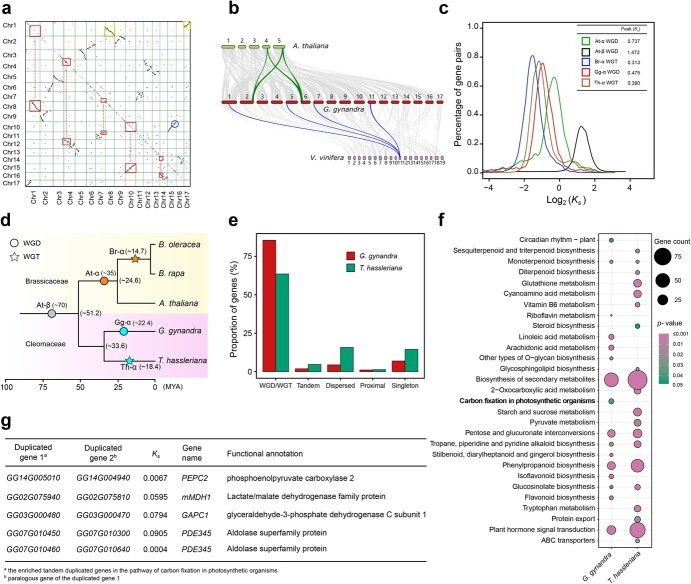
Analysis of WGD and tandem duplications in the *G. gynandra* genome. **a** Dotplot figure showing syntenic duplicates within the *G. gynandra* genome. Red squares linked by dashed lines denote the extensive collinear relationships between chromosomes of *G. gynandra* due to the WGD event. The blue circle represents an intra-chromosomal segment inversion. The big and small yellow boxes on Chr1 depict synteny with the chromosomes of Chr8 and Chr17, respectively, suggesting occurrence of potential chromosome breakage and fusion events following WGD. **b** Micro-collinearity of the *G. gynandra* genome with those of *A. thaliana* and *Vitis vinifera*. The parallel horizontal lines represent the chromosomes of the *A. thaliana*, *G. gynandra* and *V. vinifera* genomes, with the connected grey ribbons indicating syntenic blocks. The 4-to-1 collinear relationship between *G. gynandra* and *V. vinifera* is highlighted by one syntenic set in blue, with one segment in *V. vinifera* traced to four regions in *G. gynandra*. The 2-to-2 collinear relationship between *G. gynandra* and *A. thaliana* is marked by two syntenic sets in green. **c***K*_s_ distribution of syntenic orthologs from *G. gynandra*, *T. hassleriana, A. thaliana*, and *B. rapa.* The *X*- and *Y*-axes denote log_2_(*K*_s_) and the percentage of gene pairs in the syntenic blocks, respectively. The values of *K*_s_ peaks and the corresponding WGD events for each species are shown in the top right corner. **d** Evolutionary relationship and paleopolyploidization events in the five sequenced species of Brassicaceae and Cleomaceae. The paleopolyploidization and time estimation are indicated on branches of the phylogenetic tree. Yellow and pink background colors represents the Brassicaceae and Cleomaceae families, respectively. Orange and grey circles mark two WGD events, At-α and At-β, respectively, the At-β event being shared between Brassicaceae and Cleomaceae. The cyan circle and star highlight WGD (Gg-α) and WGT (Th-α) events in *G. gynandra* and *T. hassleriana*, respectively. The divergence time is listed in brackets. The time scale (MYA) is shown at bottom. **e** Gene duplication patterns in the genomes of *G. gynandra* and *T. hassleriana*. The graph indicates the proportion of genes classified by duplication mode relative to total genes of each species. The gene duplication modes were determined by MCSCANX, including singletons, dispersed, proximal, tandem, and WGD/WGT. WGD and WGT are the major modes of gene duplication for *G. gynandra* and *T. hassleriana*, respectively. **f** KEGG enrichments of the tandem duplicated genes in *G. gynandra* and *T. hassleriana*. The size of the circle indicates enriched gene numbers in each pathway, with the color of the circle indicating enrichment *P*-value. Pathways with *P*-value <.05 are shown. The ‘carbon fixation in photosynthetic organisms’ pathway is specifically enriched in *G. gynandra* (marked in bold). **g** Tandem duplicated genes of the carbon fixation pathway enriched in *G. gynandra*. Duplicated gene paralogs are listed in columns 1 and 2. *K*_s_ values were calculated using the KaKs_Calculator for duplicated gene pairs [77]. The *K*_s_ values of these genes were much lower than the *K*_s_ peak of Gg-α (0.475).

A paleopolyploidization event has been reported for the *Cleome* genus, and *T. hassleriana* underwent a whole-genome triplication (WGT) [30, 31]. However, it remains to be clarified whether this event is specific to *T. hassleriana* or shared with *G. gynandra*. We thus analyzed syntenic blocks within the *G. gynandra* genome through intra-genome comparisons, identifying 771 syntenic blocks with 8801 paralogous gene pairs (Supplementary Data Table S14). We detected multiple duplications in *G. gynandra* based on synteny, with many pairs of paralogous genes. Specifically, Chr1, Chr2, Ch3, Chr4, Chr7, Chr10, and Chr14 corresponded closely with Chr8, Chr6, Chr5, Chr12, Chr11, Chr15, and Chr16, respectively (Figs 1a and 3a). Additionally, intra-chromosomal rearrangements, especially inversions, were pervasive in *G. gynandra*, such as those near the arm ends of Chr10 and Chr15.

For inter-species comparison, we examined genomic synteny between *G. gynandra* and *A. thaliana* and *Vitis vinifera* chromosomes, resulting in a 2-to-2 syntenic relationship with *A. thaliana* and 4-to-1 with *V. vinifera* (Fig. 3b, Supplementary Data Fig. S7a and b). Of note, synteny analysis between *G. gynandra* and *T. hassleriana* showed a 2-to-3 syntenic relationship (Supplementary Data Fig. S7c). Given that *V. vinifera* has not undergone genome duplication [32] and that *A. thaliana* experienced a recent whole-genome duplication (WGD), termed At-α [33], we thus named the species-specific WGD event in *G. gynandra* and WGT event in *T. hassleriana* Gg-α and Th-α, respectively. To further infer the time of this Gg-α, we calculated the density distribution of *K*_s_ (synonymous substitution rate) and D4DTv (distance of 4-fold degenerate transversion) values of collinear gene pairs within *G. gynandra*, *T. hassleriana*, *A. thaliana*, and *B. rapa*. The distribution of *K*_s_ values showed that *G. gynandra* had one main peak at *K*_s_ of ~0.475 (~22 MYA), which was slightly earlier than *B. rapa* at ~0.313 (~14.7 MYA) and *T. hassleriana* at ~0.390 (~18.4 MYA), and later than *A. thaliana* at ~0.737 (~35 MYA; Fig. 3c and d). The distribution of D4DTv in these four species corroborated time relationships of the WGD or WGT event (Supplementary Data Fig. S7d). About 85.5% of genes in the *G. gynandra* genome were duplicated and retained from WGD, which was much higher than the proportion of 63.5% for *T. hassleriana* (Fig. 3e). Functional enrichment analysis of these WGD/WGT-derived genes identified pathways such as photosynthesis, ATP synthesis, and stress responses as markedly enriched in *G. gynandra* relative to *T. hassleriana* (Supplementary Data Fig. S8a). In addition to WGD, we also identified 610 tandem duplicated genes (TDGs), which were involved in various pathways (Fig. 3f). Of importance, the carbon fixation pathway in photosynthetic organisms was exceptionally enriched among *G. gynandra* TDGs. Their *K*_s_ values were all <0.1, indicating that these TDGs derived from the very recent duplications after Gg-α (Fig. 3g). Except for *PDE345*, the other TDGs exhibited dynamic expression levels from young to mature leaves of *G. gynandra* (Supplementary Data Fig. S8b and c). Collectively, these results revealed that, unlike C_3_*T. hassleriana*, *G. gynandra* experienced species-specific WGD (Gg-α) and tandem duplication events, both of which likely facilitated C_4_ photosynthesis evolution.

### Expanded gene families have contributed to C_4_ trait formation

**Figure 4:**
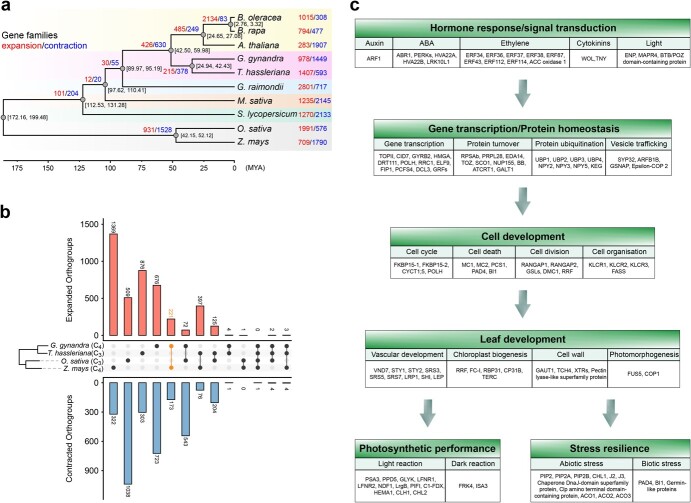
Analysis of gene family expansion and contraction in representative C_3_ and C_4_ plants. **a** Number of gene families that expanded (red) or contracted (blue) during evolution mapped to the species phylogenetic tree. The black number at each node denotes the estimated divergence time between the two branches. The time scale (MYA) is shown at bottom. **b** Numbers of expanded and contracted orthogroups shared among the C_3_ and C_4_ lineages. The middle panels show phylogenetic tree and comparison groups of the four species, *G. gynandra* (C_4_), *T. hassleriana* (C_3_), *O. sativa* (C_3_), and *Z. mays* (C_4_). Upper and lower panels indicate numbers of shared expanded and contracted orthogroups, respectively, with numbers noted for each bar. The number of expanded orthogroups shared between C_4_ plants *G. gynandra* and *Z. mays* is marked in orange (221). **c** Functional annotation of the expanded gene families common to *G. gynandra* and *Z. mays*. Arrows indicate biological processes potentially associated with the fitness advantages of C_4_ plants, including hormone response/signal transduction, gene transcription/protein homeostasis, cell development, leaf development, photosynthetic performance, and stress resilience. The genes within each biological process are divided into different categories based on their functional annotations from MapMan.

Closer inspection of gain and loss within gene families showed that 978 and 1449 families have undergone expansion and contraction in *G. gynandra*, respectively (Fig. 4a and Supplementary Data Table S15). Notably, we identified 221 expanded orthogroups, which were shared by the C_4_ dicot *G. gynandra* and C_4_ monocot *Z. mays* (Fig. 4b). These commonly enriched families could be mainly classified into six functional categories: hormone response/signal transduction, gene transcription/protein homeostasis, cell development, leaf development, photosynthetic performance, and stress resilience (Fig. 4c, Supplementary Data Fig. S9). Particularly, genes associated with cell and leaf developments were expanded in both C_4_ species. Expansion of genes in light and dark reactions could enhance the photosynthetic efficiency of C_4_ plants, and gene families involved in resistance to abiotic or biotic stress were also expanded (Fig. 4c). These results suggest that the fitness advantages of C_4_ species may have relevance to expansions of defined gene families.

### Gene expression patterns for high vein density and heat stress tolerance associated with maintenance of C_4_ photosynthesis in *G. gynandra*

**Figure 5:**
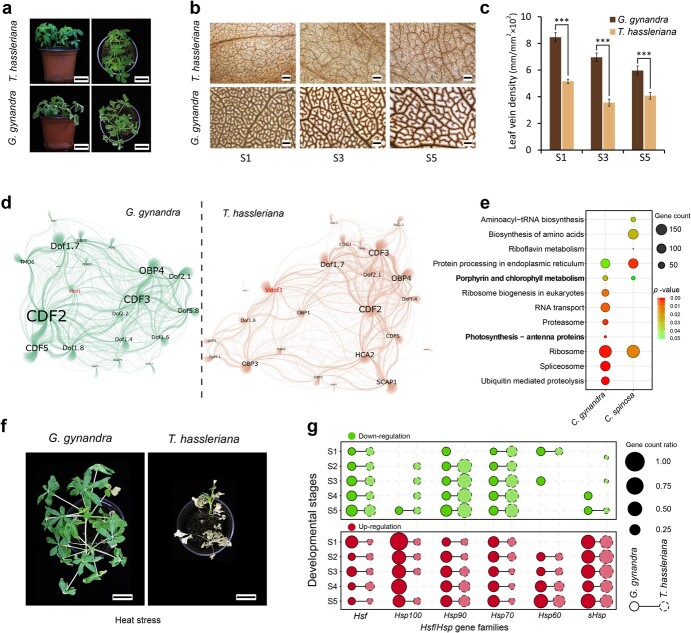
Higher leaf vein density and heat stress resistance in *G. gynandra* compared with *T. hassleriana* associated with gene expression modifications. **a** Growth phenotype of *G. gynandra* (lower panel) and *T. hassleriana* (upper panel) under normal conditions. The photographs are of 5-week-old plants. The left and right panels are side and top views, respectively. Scale bar = 5 cm. **b** Overview of leaf vein patterns in *G. gynandra* (bottom) and *T. hassleriana* (top). Representative images from left to right illustrate vein density of the central region at leaf developmental stages from young to mature as numbered (S1, S3, S5). Scale bar = 200 μm. **c** Analysis of leaf vein density between *G. gynandra* and *T. hassleriana.* Vein density (vein length/area) for each leaf was assessed for six positions at a central region bounded by the midvein over three developmental stages. Student’s *t*-test (mean ± standard deviation; *n* = 3); ^***^P < .001. **d** Networks of gene regulatory relationships between Dof transcription factors and their candidate target genes in *G. gynandra* and *T. hassleriana*. GRNs were constructed using GENIE3 and visualized with Gephi. The size of the labeled hub gene names is based on the degree of connecting nodes. Edges of *G. gynandra* (left) and *T. hassleriana* (right) GRNs are indicated in green and tangerine colors, respectively. The *Vdof1* gene in the two GRNs is highlighted in red. **e** KEGG pathway enrichment of candidate target genes of the Vdof1 transcription factor in *G. gynandra* and *T. hassleriana*. The size of the circle indicates enriched gene numbers in each pathway, with the color of the circle indicating enrichment *P*-value. Pathways with *P*-value <.05 are shown. Photosynthesis-related pathways are marked in bold. **f***Gynandropsis gynandra* displays exceptional tolerance to continuous high temperature stress compared with *T. hassleriana*. Five-week-old plants were subjected to heat treatments in a controlled growth chamber under conditions of 45/35°C (16/8 h) and 60% relative humidity for 15 days. Scale bar = 5 cm. **g** Proportions of up- and downregulated genes for *Hsf*/*Hsp* families after heat treatments in *G. gynandra* and *T. hassleriana*. The *X*- and *Y*-axes indicate *Hsf*/*Hsp* family names and different leaf developmental stages (from young to mature, S1 to S5), respectively. The size of the circle denotes the ratio of differentially expressed gene count relative to the total gene number of each *Hsf*/*Hsp* family. The lower (red) panel depicts the proportions of upregulated genes, and the upper (green) panel shows the proportions of downregulated genes.

*Gynandropsis gynandra* and *T. hassleriana* exhibited similar phenotypes regarding morphology and leaf development (Fig. 5a, Supplementary Data Fig. S10). However, *G. gynandra* possessed evidently denser veins (Fig. 5b) and typical Kranz anatomy compared with *T. hassleriana* (Supplementary Data Fig. S11). To identify potential discrepant features between these dicotyledonous C_3_ and C_4_ plants, leaf samples from five developmental stages were collected under normal and heat stress conditions (Supplementary Data Fig. S11). We found that *G. gynandra* had significantly higher vein density than *T. hassleriana* during early (S1), middle (S3), and mature (S5) stages (Fig. 5c). Many of the well-known genes encoding vasculature developmental factors had differential expression patterns between the two species (Supplementary Data Fig. S12). To dissect mechanisms underlying the differences in transcriptional regulation of the leaf vasculature, we constructed large-scale gene regulatory networks (GRNs) based on RNA-seq time series datasets, and candidate target genes of transcription factors were then predicted (Supplementary Data Fig. S13). Among many of the transcription factors, the Dof family is established to be enriched in bundle sheath cells and binds to AAAG motifs [34]. Twenty-one and 24 hub genes from Dof-GRNs were identified for *G. gynandra* and *T. hassleriana*, respectively (Fig. 5d). In addition to the well-documented *Dof* genes with important roles in vein development, we also found some *Dof* genes with unknown functions (Supplementary Data Table S16). By analyzing the previously deposited cell-specific transcriptomic data [35], we found that both homologous genes of *Vdof1* in *G. gynandra* exhibited preferential expression in bundle sheath cells (Supplementary Data Fig. S14). The potential target genes of *G. gynandra Vdof1* were shown to be mainly enriched in pathways for chlorophyll metabolism, photosynthesis, and RNA and protein metabolism (Fig. 5e). Overexpression of *Vdof1* conferred significantly higher leaf vein density in *Arabidopsis* plants (Supplementary Data Fig. S15).

*Gynandropsis gynandra* was much more tolerant to high temperature than *T. hassleriana* (Fig. 5f). We then analyzed differential gene expression in the two species (Supplementary Data Table S17). The heat stress response pathway was significantly upregulated at all stages in *G. gynandra* but not in *T. hassleriana* (Supplementary Data Fig. S16). A set of *Hsf* and *Hsp* was identified in both species (Supplementary Data Table S18). However, 12–40% of *Hsf* genes in *G. gynandra* exhibited significantly upregulated expression after heat treatments, compared with 7–19% for *T. hassleriana* (Fig. 5g). Consistently, the photosynthesis pathway was over-represented among the heat-upregulated genes of *G. gynandra*, regardless of developmental stage (Supplementary Data Fig. S16), including a subset of C_4_ metabolism-related genes (Supplementary Data Fig. S17), while differential expression patterns were observed for photorespiration pathway genes between *G. gynandra* and *T. hassleriana*.

### Expression features and evolution of key genes involved in C_4_ photosynthesis and photorespiration

**Figure 6:**
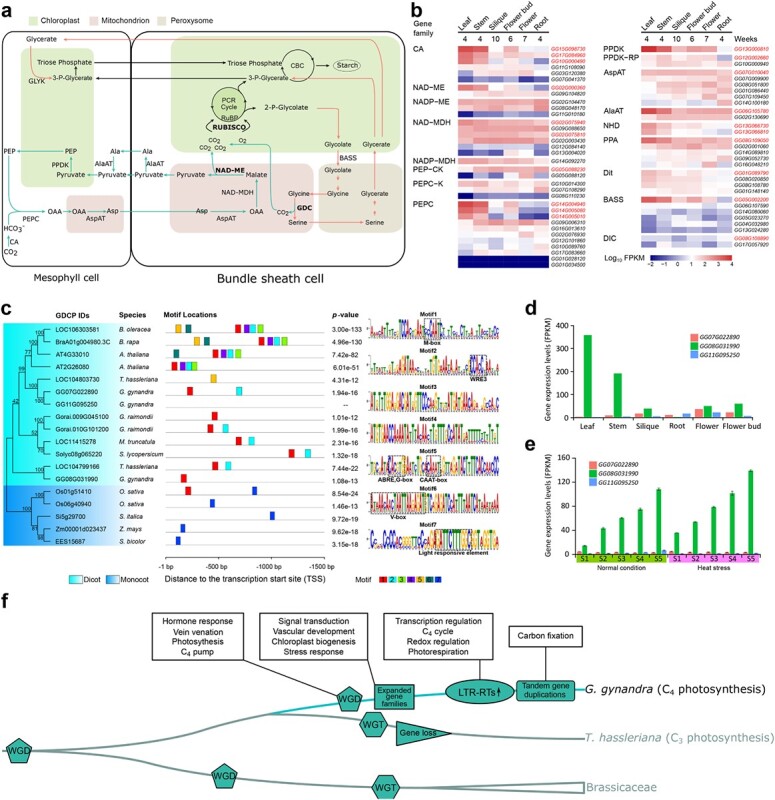
Evolution of C_4_ photosynthesis in *G. gynandra*. **a** Diagrammatic representation of main proteins and metabolic fluxes for the NAD-ME C_4_ photosynthetic subtype in *G. gynandra*. Aspartate (Asp) converted from oxaloacetate (OAA) by AspAT in the mitochondria of mesophyll (M) cells is the main metabolite transported from M cells to bundle sheath cells. Asp is converted to OAA and then to malate (Mal), which is decarboxylated by NAD-dependent malate dehydrogenase (NAD-MDH); Mal is further decarboxylated by NAD-ME, releasing CO_2_ to the chloroplast of bundle sheath cells. The green arrows show the carbon dioxide (CO_2_) accumulation pathway of NAD-ME subtype. Red arrows mark the photorespiratory pathway. The three key enzymes, RuBisCO, NAD-ME and GDC, are in bold. Full names of metabolites and enzyme abbreviations are listed in Supplementary Data Table 21. PCR cycle, photosynthetic carbon reduction cycle; CBC, Calvin–Benson cycle. **b** Heat map showing the expression pattern of key candidate genes involved in C_4_ photosynthesis in photosynthetic and non-photosynthetic tissues of *G. gynandra*. The color denotes the expression level from low (blue) to high (red) expressed as log_10_FPKM. **c** Phylogenetic tree and motifs in the promoter regions of *GDCP* genes in representative C_3_ and C_4_ plants. The left panel shows the phylogenetic tree of GDCPs, with light blue and dark blue backgrounds marking dicot and monocot species, respectively. The middle panel shows the type, location, and *P*-values of conserved motifs in the upstream 1.5-kb regions from the transcription start site of each *GDCP* gene. The right panel shows the motif sequence and *cis*-acting regulatory elements highlighted by dashed boxes. The scale length (bp) and motif color key are shown at the bottom. **d** Expression patterns of the three *GDCP* genes in different tissues of *G. gynandra*. Unique among the paralogs, the *GG08G031990* gene showed a very high level of expression in the leaf or stem. **e** Analysis of expression levels of the three *GDCP* genes during *G. gynandra* leaf development under normal or heat stress conditions*.* The *X*- and *Y*-axes indicate gene expression level (FPKM) and leaf developmental stages (from young to mature, S1–S5), respectively. Consistent with the results in panel **d**, only *CG08G031990* was dominantly expressed in each stage analyzed, and its expression was even inducible by heat. **f** Conceptual model reconstructing hypothesized key steps in the evolution and maintenance of C_4_ photosynthesis in *G. gynandra*. First, a recent WGD (Gg-α) is the origin of C_4_ pathway-related genes in *G. gynandra*, including genes involved in the hormone response, venation, C_4_ metabolism, Calvin cycle, N and S assimilations, and photorespiratory CO_2_ pump. Second, expansions of defined gene families enable the establishment of C_4_ features including signal transduction, vascular development, chloroplast biogenesis, and stress response. Finally, more recent LTR-RT explosion and species-specific tandem duplications fine-tune the expression of C_4_ photosynthesis-associated genes, including those involved in the light reaction, C_4_ cycle, redox regulation, and carbon fixation. These steps can overlap and may not have taken place independently. The much higher leaf vein density and heat stress tolerance in *G. gynandra* compared with *T. hassleriana* are linked to shifted patterns of gene expression. Despite *T. hassleriana* having undergone a WGT (Th-α) event later than Gg-α, subsequent massive gene loss likely occurred, including the missing duplicated copies of certain C_4_ metabolism genes. Besides, contrary to the photorespiration pathway, the overall expression levels of the C_4_ pathway-related genes in *T. hassleriana* are markedly lower than in *G. gynandra*, which might have also constrained its evolution of C_4_ photosynthesis. Brassicaceae and Cleomaceae shared a WGD (At-β) event before divergence, and then Brassicaceae underwent another WGD (At-α) event, with *Brassica* species further experiencing an extra WGT (Br-α) event.

An illustration of the NAD-ME subtype of C_4_ photosynthesis is shown in Fig. 6a. Targeted analysis of the copy number of C_4_ cycle genes revealed that the ratio of gene copy number in *G. gynandra* versus *T. hassleriana* was mostly higher than 2:3, which is the baseline expected ratio given their genomic relationship (Supplementary Data Fig. S18). This suggests that *G. gynandra* likely retained more C_4_ genes after WGD, whereas *T. hassleriana* could have lost a subset of these genes after WGT (Supplementary Data Table S18). All the enzymes characterizing the NAD-ME subtype were identified in *G. gynandra* (Fig. 6b, Supplementary Data Table S18). C_4_ enzyme genes are preferentially expressed in photosynthetic tissues. Furthermore, candidate homologous enzymes involved in C_4_ carbon fixation in *G. gynandra* were identified based on their preferential expression in photosynthetic tissues and phylogenetic analysis with known C_4_ genes (Supplementary Data Fig. S19).

Interestingly, three copies of the *GDCP* gene were detected in *G. gynandra*, while a single copy was present in C_4_ monocots (*S. bicolor*, *S. italica*, and *Z. mays*) and one or two copies were present in C_3_ plants (Fig. 6c). Unexpectedly, M-box, but not V-box, was present in the promoters of two *GDCP* genes in *G. gynandra*, and no known motifs could be detected in the other one. It should be mentioned that neither M-box nor V-box was found in the *GDCP* of C_4_ monocots. Notably, a single *GG08G031990* was the most abundantly expressed in *G. gynandra*, with the highest levels in leaf tissue, while the other two duplicates were expressed at extremely low levels in all tissues analyzed (Fig. 6d). This paralog was uniquely increased along leaf developmental gradients and could be further dramatically induced by heat stress (Fig. 6e). Based on our present study, we propose a conceptual model of C_4_ evolution in the *Cleome* genus (Fig. 6f). A three-step scenario may contribute to the evolution of C_4_ photosynthesis. Innovation of this novel biochemical pathway in *G. gynandra* could be dual consequences of duplicated gene retention and functional changes in existing genes. Specifically, a recent WGD (Gg-α) event is the origin of C_4_ metabolism-related genes, followed by corresponding gene family expansions that facilitate C_4_ feature establishment. Then more recent LTR-RT bursts and tandem duplications further fine-tune the expression of C_4_ photosynthesis genes in *G. Gynandra*. Although *T. hassleriana* experienced a WGT (Th-α) event, massive loss of copies of C_4_ pathway genes happened, which limited its formation of a C_4_ cycle (Fig. 6f).

## Discussion

The leafy vegetable *G. gynandra* has been an instrumental organism for addressing evolutionary questions, often as a pair with the ornamental horticulture plant *T. hassleriana*. This species is valuable for the study of its nutritional and health-promoting properties, as well as molecular mechanisms absent in C_3_ model organisms such as the dicot *Arabidopsis* and the monocot rice, and represents a model plant to extensively investigate C_4_ biology [14, 26, 27]. The lack of a telomere-to-telomere gapless chromosome-scale genome of *G. gynandra* has hampered its wide adoption in basic and applied research, while the recently released *G. gynandra* genome only has a contig N50 of 24.3 kb, LAI of ~0, and assembled genome percentage of 79.5%, without telomere-to-telomere coverage but with 73 966 gaps on the total 17 pseudochromosomes (Supplementary Data Table S21) [36]. Here we have generated a telomere-to-telomere reference-grade genome of *G. gynandra* integrating long-read nanopore sequencing, PacBio HiFi, and Hi-C technologies to overcome the challenge inherent in highly repetitive sequences of this species (Figs 1 and 2). With a contig N50 of 11.43 Mb, LAI of 16.46, assembled genome percentage of 98.66%, 10 gap-free chromosomes, and only 11 gaps on the entire *G. gynandra* genome, these assembly metrics demonstrate high accuracy, continuity, and completeness (Table 1, Supplementary Data Tables S5 and S21). Genomics combined with transcriptomics analysis confirmed that *G. gynandra* is the typical NAD-ME subtype of C_4_ plants (Fig. 6). Given that an *Agrobacterium tumefaciens*-mediated transformation system with high efficiency has also been established for this elite crop [21], *G. gynandra* is now suitable as a C_4_ model system in dicotyledonous plants, particularly for studies in the NAD-ME subtype of C_4_ species, such as the perennial polyploid bioenergy crop switchgrass (*Panicum virgatum*).

The evolution of plant C_4_ photosynthesis represents a unique example of convergent evolution [4, 37, 38]. With the availability of the annotated genomes for *G. gynandra* and *T. hassleriana*, direct comparison between these C_4_ and C_3_ species presents an excellent model to unravel why C_4_ photosynthesis evolved in only one of the two plants. Instead of sharing a Th-α event [31], these two species experienced independent recent WGD (Gg-α) and WGT (Th-α) events, respectively (Fig. 3). However, massive gene loss may have happened in C_3_*T. hassleriana* after Th-α (Supplementary Data Fig. S18, Supplementary Data Table S19), which resulted in retention of only 27 396 predicted genes, making *T. hassleriana* the gene-poorest species of those analyzed here (Fig. 1d). Of note, the copy numbers of multiple C_4_ pathway-related genes are higher in *G. gynandra* relative to *T. hassleriana* (Supplementary Data Table S18), concomitant with markedly reduced expressions of vasculature development and C_4_ metabolism genes in *T. hassleriana* (Supplementary Data Fig. S20). Peculiarly, *G. gynandra* has retained the paralogous genes essential for building the photorespiratory CO_2_ pump in bundle sheath cells (Supplementary Data Tables S18 and S20) [39], whose formation is hypothesized to be the primary driver of C_4_ evolution [5, 40]. These data suggest that *G. gynandra* underwent the C_3_–C_4_ intermediate stage required to establish the C_4_ pathway.

Heat, intense light, and/or drought stress were proposed to drive C_4_ origination and evolution during periods of declining atmospheric CO_2_, suggesting the role of adaptive evolution in C_4_ pathway formation [4, 41]. The spider flower *G. gynandra* displays much higher tolerance to extreme temperature than *T. hassleriana*, and this phenotype is likely associated with retention of a subset of WGD-duplicated genes in *G. gynandra* that encode heat shock factors (HSFs) and heat shock proteins (HSPs) (Fig. 5f, Supplementary Data Table S20). Although their expressions can be induced under heat stress in both species, many more of these families are upregulated in *G. gynandra* compared with *T. hassleriana* (Fig. 5g). Given that C_4_ photosynthesis in *G. gynandra* evolved after WGD [31], this provides additional evidence on the critical role of Gg-α in facilitating C_4_ evolution.

Aside from WGD events and the expanded gene families, recent species-specific tandem duplications after Gg-α may also be relevant to maintenance of the C_4_ pathway in *G. gynandra*. Remarkably, our data unveiled a previously undescribed role for genomic transposon expansion in the evolution of C_4_ photosynthesis. LTR-RT activity might have promoted the C_3_-to-C_4_ evolution via synergistic genomic and transcriptomic alterations. Consistent with the recent reports demonstrating that modifications in *cis-*regulatory and non-coding regions are primary determinants of the distinct transcriptome blueprint in bundle sheath cells or mesophyll cells of C_4_ plants [34, 42, 43], this extensive LTR-RT insertion frequency in promoter regions appears to be an underappreciated route through which cell-preferential gene expression could be achieved. Future molecular characterization of regulatory regions containing LTR-RTs will be required to test this hypothesis.

In summary, we have generated a highly continuous and accurate genome assembly of the ornamental horticulture crop *G. gynandra*. These findings shed light on the commonalities and differences in the evolution of C_4_ photosynthesis, supporting the existence of numerous independent evolutionary trajectories to C_4_. The genomic and transcriptomic data generated in this study provide valuable new resources for further dissecting the genetic basis underlying the transition from C_3_ to C_4_ photosynthesis as well as the exceptional nutritional and medicinal traits of this species. *Gynandropsis gynandra* is thus now a promising model species to accelerate both basic and applied research, in C_4_ biology and beyond.

## Materials and methods

### Plant materials and growth conditions

C_4_*G. gynandra* Linn. and C_3_*T. hassleriana* (Purple Queen) species were grown in plastic pots with Pro-Mix BX soil, in a controlled environment room with a photoperiod of 16 h/8 h, a light intensity of 200 μmol m^−2^ s^−1^, 25/18°C (day/night), and a humidity of 50–60%. Leaves (4 weeks), stems (4 weeks), roots (4 weeks), flower buds (6 weeks), flowers (7 weeks), and siliques (10 weeks) of plants were harvested and immediately frozen in liquid nitrogen.

### Heat treatment

Five-week-old plants of both species were transferred to a growth chamber (Percival E-41, USA) using the conditions above, except that the day/night temperature was set to 45/35°C, which is 10–15°C above the optimum range of germination for *G. gynandra* and is considered as heat stress [44]. Leaves at different developmental stages were sampled from plants at 10.00 a.m. before and after heat treatment. Meanwhile, leaf samples at the equivalent stages under normal growth conditions were collected as controls (Supplementary Data Fig. S10). Stages 1–5 (S1–S5) were leaves from young to mature, with S1 as the youngest (1.5 cm in length). All experiments were performed in triplicate.

### Genome sequencing

Nanopore, Illumina HiSeq and Hi-C were used for sequencing the complete genome of *G. gynandra*. For Illumina sequencing library construction, genomic DNA of *G. gynandra* was extracted from young leaves using a Qiagen DNA purification kit (Qiagen, Darmstadt, Germany). The integrity of DNA was assessed by agarose gel electrophoresis, and the purity and concentration were determined with a NanoDrop 2000 spectrophotometer (Thermo Fisher Scientific, MA, USA) and a Qubit Fluorometer (Invitrogen, Carlsbad, CA, USA), respectively. The short-read genomic sequencing libraries were constructed using the MGIEasy FS DNA Library Prep Set (Item No. 1000006988) with 270 bp fragment size according to the manufacturer’s instructions, and sequenced on an Illumina HiSeq 4000 platform (Illumina, San Diego, CA) to generate paired-end reads of 150 bp. For nanopore sequencing library preparation, high-quality genomic DNA of *G. gynandra* was extracted from young leaves using the CTAB method. Approximately 15 μg of genomic DNA was used to collect larger DNA fragments (>20 kb) with the BluePippin Size-Selection System (Sage Science, Beverly, MA, USA). The obtained genomic DNA was processed using an ONT Ligation Sequencing Kit (SQK-LSK109; Oxford Nanopore Technologies, Oxford, UK) according to the manufacturer’s instructions. Briefly, the genomic DNA was end-repaired and dA-tailed using the NEBNext Ultra II End Prep Reaction Module (New England Biolabs, Ipswich, MA, USA). The sequencing adaptors were ligated using the NEBNext Ultra II Ligation Module (New England Biolabs, Ipswich, MA, USA). Libraries were purified using AMPure XP beads (Beckman Coulter, CA, USA) and short fragment buffer (SFB). Then, nanopore libraries were added into a single ONT MinION R9.4 flowcell (FLO-MIN106) and sequenced on the platform of PromethION (Oxford Nanopore Technologies, UK). The ONT MinKNOW software (https://github.com/nanoporetech/minknow_lims_interface.git) acquired raw sequence data with live base-calling by ONT Guppy (https://nanoporetech.com/). For Hi-C sequencing, the fresh leaf tissues of *G. gynandra* were fixed (cross-linking) with formaldehyde. The cross-linked chromatin was extracted and digested using the MboI restriction enzyme, and the 5′ overhangs were filled in with biotinylated nucleotides and blunt-end proximity-ligated to generate circular molecules. Subsequently, the circular DNA molecules were purified from protein, and sheared to ~350 bp mean fragment size, and then enriched by biotin pull-down. The sequencing libraries were generated using NEBNext Ultra enzymes (New England Biolabs, Ipswich, MA, USA) and Illumina-compatible adapters (Illumina, San Diego, CA, USA). The Hi-C libraries were processed to paired-end sequencing on the Illumina HiSeq 4000 (Illumina, San Diego, CA, USA) platform with read length of 150 bp.

### Genome size estimation

The genome size of *G. gynandra* was estimated using *k*-mer analysis. Raw reads from Illumina sequencing were subjected to SOAPnuke (https://github.com/BGI-flexlab/SOAPnuke) for base quality control. The genome size, abundance of repetitive elements, and heterozygosity were estimated based on the *k*-mer frequencies generated from the short reads using GenomeScope (https://github.com/schatzlab/genomescope).

### *De novo* genome assembly

The ONT long reads were corrected and assembled using CANU v1.7.1 with the parameters (corOutCoverage = 50, saveOverlaps = TRUE, ovsMemory = 64, minMemory = 30G, batMemory = 200G, minOverlapLength = 700, minReadLength = 1000) [45]. The resulting contigs were polished with both long reads and short reads using three rounds of Racon v1.4.13 with default parameters [46]. The Illumina short reads were mapped onto the polished assembly with BWA-MEM (https://github.com/bwa-mem2/bwa-mem2). Based on the alignment, error correction was conducted using Pilon v1.23 [47]. To scaffold the assembled contigs, the ONT-based polished contigs were anchored into a chromosome-scale assembly using a Hi-C proximity-based assembly approach. Illumina reads from the Hi-C library were processed with SOAPnuke to remove adaptor and low-quality sequences. The clean Hi-C read pairs were used as input for the Juicer v1.6 and 3d-DNA Hi-C analysis and scaffolding pipelines v180922 [48, 49]. Valid interaction pairs were mapped onto the polished contigs and anchored to the pseudochromosomes using the 3D-DNA pipeline with default parameters. The Hi-C interaction matrix was visualized using Juicebox Assembly Tools, and mis-assemblies and mis-joins were manually corrected based on neighboring interactions. Raw data produced by the PacBio Sequel II platform (Pacific Biosciences; http://www.pacb.com) were processed through the SMRT Analysis software suite (v5.1.0). The consensus HiFi reads were produced by the CCS subprogram (https://github.com/PacificBiosciences/ccs) with default parameters. The low-quality reads and sequence adapters were removed to obtain clean subreads. The long (~15 kb) and highly accurate (>99%) HiFi reads were assembled using hifiasm (0.19.4) with default parameters. The corrected HiFi reads along with the corrected ONT reads were used for gap filling, generating the final pseudochromosome-length telomere-to-telomere genome assembly.

### Genome assembly quality assessment

Three assessment strategies, including BUSCO alignment, transcriptome alignment, and LAI, were used to evaluate the quality and completeness of the *G. gynandra* genome assembly. In brief, the completeness of the genome assembly was evaluated using the BUSCO pipeline based on the datasets of embryophyta_odb10. The coverage and base-level accuracy of the genome assembly were assessed by aligning transcriptome reads to the *G. gynandra* assembly using HISAT2 with default parameters. The LAI score of the *G. gynandra* genome assembly was calculated using LTR_retriever v2.9.0 with default parameters [50].

### Genome annotation

The *G. gynandra* genome was annotated including annotations of repeat sequences, protein-coding genes and RNA genes. First, we adopted two complementary methods (one homology-based and the other *de novo*-based) to predict repeat sequences. RepeatMasker v4.0.7 and RepeatProteinMask (http://www.repeatmasker.org) were used to discover and classify repetitive sequences with the homology-based library generated from Repbase (www.girinst.org/repbase) [51]. LTR_FINDER v1.06 and RepeatModeler (http://www.repeatmasker.org/RepeatModeler) were utilized to build the *de novo*-based library [52]. TRF v4.09 was employed to annotate tandem repeats with default parameters [53]. Full length LTR-RTs were identified using LTR_retriever. Second, we deployed a strategy combining *ab initio*, homology-based, and transcriptome-based methods for gene structure annotation. We used both Augustus v3.3.2 and SNAP v1.0.4 to perform *ab initio* predictions with self-trained prediction models [54, 55]. We aligned the protein sequences from *A. thaliana*, *B. oleracea*, *B. rapa*, *T. hassleriana*, *G*. *raimondii*, *M. sativa*, *S. lycopersicum*, *O. sativa*, and *Z. mays* to the repeat-masked genome of *G. gynandra*, and then parsed the resultant alignments by GeneWise (https://www.ebi.ac.uk/~birney/wise2/) to achieve homolog predictions. We mapped the transcripts generated from both PacBio Iso-Seq and Illumina RNA-seq to the genome with PASA vr20140407 to conduct transcriptome-based predictions [56]. Finally, we combined all the evidences to finalize the consensus gene models using Maker v2.31.9 [57]. Third, we performed RNA gene annotation by tRNAscan-SE v2.0 for tRNA [58], RNAmmer v1.2 for rRNA [59], and INFERNAL v1.1.2 for miRNA and snRNA [60]. In addition, we annotated transcription factor encoding genes using iTAK v1.7 [61]. We generated functional assignments of protein coding genes by performing BLAST searches against five public protein databases including the NCBI non-redundant (nr) database, Swiss-Prot database, the Clusters of Orthologous Genes (COG) database, the Kyoto Encyclopedia of Genes and Genomes (KEGG) database, and the Gene Ontology (GO) database. Pfam domains of genes were identified using InterProScan.

### Phylogenomic evolution analysis

Orthology prediction for 10 species, including 8 eudicots and 2 monocots, was performed using the OrthoFinder v2.2.6 package with default parameters [62]. Single-copy orthologous genes were extracted from the clustering results, and were used to reconstruct the phylogenetic tree. In brief, the proteins of single-copy gene families were aligned by MUSCLE v3.8.31 [63]. The alignments were finally joined into a supergene matrix for the species phylogenetic tree construction using RAxML v8.2.12 [64] with the JTT + I + GAMMA model and 1000 bootstrap replicates. The best suitable evolution model for phylogeny construction was evaluated using jModelTest v2.1.10 [65]. Time estimation among species was performed with r8s v1.81 [66]. The divergence times of *B. oleracea* and *B. rapa* (2.02–3.21 MYA), *Z. mays* and *O. sativa* (42–52 MYA), and *A. thaliana* and *O. sativa* (115–308 MYA) obtained from TimeTree (http://timetree.org) were used for calibration. Syntenic blocks and gene duplications were identified within the *G. gynandra* genome or between the *Cleome* plants and other species using MCScanX v0.8 with the parameters (−s 5, −m 5) [67]. The synonymous mutation rate values for gene pairs within syntenic blocks were calculated using PAML v4.9 with yn00 and NG model [68]. Four-fold degenerative transversion (4DTv) rates were calculated by aligning all orthologous or paralogous gene pairs using an in-house Perl script. We identified the WGD event within the *G. gynandra* genome using the approach from PGDD [69]. CAFE v4.2.1 was used to identify the gene families with rapid expansion or contraction based on the species phylogenetic tree and divergence time [70]. The species-specific gene families were determined according to the presence and absence of genes for specific species.

### RNA-seq analysis

For RNA-seq experiments, total RNAs were extracted and purified from each collected sample using the Trizol RNA extraction kit (Invitrogen, Carlsbad, CA, USA). The mRNA was enriched from ~50 ng of high-quality total RNA with NEXTflex™ Poly(A) Beads (Bioo Scientific, Austin, TX, USA), and used to produce RNA-seq libraries with the NEBNext Ultra RNA Library Prep Kit for Illumina (New England Biolabs, Ipswich, MA, USA) according to the manufacturer’s protocol. The RNA-seq libraries were sequenced with Illumina HiSeq 4000 (Illumina, San Diego, CA, USA) under PE150 mode. The raw reads of RNA-seq were filtered using Trimmomatic v0.40 with default parameters [71]. The remaining clean reads were mapped to the genome using HISAT2 v2.1.0 [72]. The read count per gene was calculated with HTSeq v0.9.172 [73]. Differentially expressed genes (DEGs) were identified using the DEseq2 package with a significance threshold of *q* value <0.01 and |log_2_(fold change)| > 1 [74].

### Iso-Seq analysis

Total RNAs extracted from different tissues collected as described above were combined to generate a full RNA sample of *G. gynandra*. The mRNA was enriched from total RNA using a magnetic d(T) bead binding procedure, and then was transcribed to cDNA using the Clontech SMARTer PCR cDNA Synthesis Kit (Clontech, Mountain View, CA, USA) following the manufacturer’s instructions. The amplified cDNA fragments were size-selected using a BluePippin Size Selection System (Sage Science, MA, USA) with a bin of >4 kb. The amplified and size-selected cDNA products were used to generate SMRTbell libraries according to the Iso-Seq protocol (P/N100377-100-05 and P/N100-377-100-04). The libraries were prepared for sequencing by annealing a sequencing primer and binding polymerase to the SMRTbell templates using the DNA/Polymerase Binding Kit (Pacific Biosciences, Menlo Park, CA, USA). One SMRT cell was sequenced on the PacBio Sequel instrument (Pacific Biosciences, Menlo Park, CA, USA). High-quality, full-length, and consistent transcript sequences were obtained from long read data using the PacBio Iso-Seq3 pipeline.

### Phylogenetic and *cis*-acting regulatory element analysis

The protein sequences of *GDCP* genes were used to perform multiple sequence alignment using MUSCLE (Supplementary Data Table S22). The phylogenetic tree was inferred using the neighbor-joining (NJ) method available in MEGA7 v7.0.26 [75]. The robustness of each node in the tree was determined using 1000 bootstrap replicates. The upstream 1.5-kb regions from the transcription start site (TSS) of each *GDCP* gene were extracted from the genome sequence of each species, and used to identify conserved motifs using MEME and *cis*-acting regulatory elements by PlantCARE server (http://bioinformatics.psb.ugent.be/webtools/plantcare/html).

### Analysis of leaf anatomy

Leaf samples were rinsed twice in water and placed in 70% ethyl alcohol for 3–4 days. The 70% ethyl alcohol was changed every 8 h until the leaves became colorless and transparent. Leaves were rinsed again with water and mounted in 66% glycerol on slides for observation. Images of the cleared leaves were taken with an Olympus BX43 microscope system equipped with an Olympus DP74 camera at 2× magnification (Olympus, Tokyo, Japan). Vein length and area size were assessed for six sites at a central region of each leaf bounded by the midvein using phenoVein tools developed from MeVisLab (www.mevislab.de) [76]. Vein density (Vd) was calculated as the total length of all veins within the region divided by the area of the region, and was expressed in millimeters per square millimeter. Measurements were conducted with three independent leaves of each stage per species.

## Supplementary Material

Web_Material_uhad129

## Data Availability

All sequence data has been deposited in the Genome Sequence Archive (GSA) (https://bigd.big.ac.cn/gsa). Genomic data: *G. gynandra*-PRJCA017363; transcriptomic data: *G. gynandra*-PRJCA017364; *Tarenaya hassleriana*-PRJCA017365. The chromosome-scale annotated genome assembly of *G. gynandra* is available at the Genome Warehouse in the National Genomics Data Center (NGDC) (https://ngdc.cncb.ac.cn) under accession number GWHCBIX00000000.
