# Supplementary material for: Integrated global analysis in spider flowers illuminates features underlying the evolution and maintenance of C4 photosynthesis
Source: Hortic Res. 2023 Jun 20;10(8):uhad129. doi: 10.1093/hr/uhad129 (PMC10407600; doi:10.1093/hr/uhad129)
Supplement: Web_Material_uhad129 [file web_material_uhad129.zip › Supplementary Material for HR.pdf]

Supplementary Material

Integrated global analysis in spider-flowers illuminates features underlying the evolution and maintenance of C_4 photosynthesis

Wei Zhao[1,†], Jun Li[1,†], Xingchao Sun[1], Qiwei Zheng[1], Wei Hua[1,2,*] and Jun Liu[1,*]

Figure S1 Statistics for the frequencies and depths of 15 *k*-mers in the *G. gynandra* genome.

Figure S2 Overview of the processing pipeline for assembling the *G. gynandra* genome.

Figure S3 Length distribution of ONT sequencing reads.

Figure S4 KEGG functional enrichment analysis of gene families specific to *G. gynandra*.

Figure S5 The density distribution of intact LTRs, *Gypsy* and *Copia* in each chromosome of *G. gynandra*.

Figure S6 Genomic distribution of LTR-RTs and their impacts on expression patterns of the C_4 pathway-associated genes in *G. gynandra* compared to *T. hassleriana*.

Figure S7 Comparative genomic analysis of the WGD event in *G. gynandra*.

Figure S8 Functional enrichment of WGD-derived genes and expression analysis of TD-derived genes in *G. gynandra*.

Figure S9 MapMan-Bin enrichment analysis of 221 expanded gene families common to *G. gynandra* and *Z. mays*.

Figure S10 The morphology of leaves at different developmental stages for *G. gynandra* and *T. hassleriana*.

Figure S11 Representative anatomy of the leaf cross-section of *G. gynandra* and *T. hassleriana*.

Figure S12 The expression patterns of leaf and vasculature-related regulatory genes along leaf development gradients for *G. gynandra* and *T. hassleriana*.

Figure S13 The gene regulatory networks (GRNs) between transcription factors and their candidate target genes in *G. gynandra* or *T. hassleriana*.

Figure S14 Enrichment of *Vdof1* gene in leaf BS cells.

Figure S15 Overexpression of *Vdof1* gene led to leaf vein density.

Figure S16 MapMan-Bin enrichment analysis of DEGs at different stages of leaves from *G. gynandra* and *T. hassleriana* subjected to heat stress.

Figure S17 Heatmap showing expression patterns of C_4 photosynthesis-related pathway genes under heat stress in *G. gynandra* and *T. hassleriana*.

Figure S18 Copy number ratio of genes for C_4 photosynthesis-related pathways in *G. gynandra* as compared to *T. hassleriana*.

Figure S19 Phylogenetic trees of C_4 photosynthesis-related genes.

Figure S20 The expression levels of C_4 pathway-related genes with lower copy numbers in *T. hassleriana* than in *G. gynandra* along leaf development gradients.

Table S1 Estimate of *G. gynandra* genome size.

Table S2 The evaluation of ONT sequencing data for *G. gynandra*.

Table S3 Statistics of Hi-C and HiFi data for *G. gynandra*.

Table S4 Summary of *G. gynandra* genome assembly.

Table S5 Summary of *G. gynandra* seventeen pseudo-chromosomes.

Table S6 BUSCO analysis of *G. gynandra* genome assembly.

Table S7 RNA-seq data mapping summary.

Table S8 Functional annotation of the predicted genes in the assembly of *G. gynandra* genome.

Table S9 Statistics of the annotated non-coding RNAs.

Table S10 Gene numbers of transcription factor family among *G. gynandra* and nine other species.

Table S11 Numbers of gene families among *G. gynandra* and nine other species.

Table S12 Summary of repeat DNA in *G. gynandra* and *T. hassleriana*.

Table S13 List of gene IDs and their RPKM values in leaf transcriptomes from S0 to S5 developmental stages of *G. gynandra* and *T. hassleriana*.

Table S14 Syntenic blocks of *G. gynandra*.

Table S15 Pfam annotation of the expanded genes families in *G. gynandra* genome.

Table S16 The hub genes of *Dof*-GRNs of *G. gynandra* and *T. hassleriana*.

Table S17 Summary of differentially expressed genes after heat stress treatment at different leaf stages in *G. gynandra* and *T. hassleriana*.

Table S18 Copy numbers of genes that regulate vasculature development, C_4 photosynthesis and heat shock response in *G. gynandra* compared to *T. hassleriana*.

Table S19 Statistics analysis suggesting that *T. hassleriana* may have undergone massive gene loss compared to *G. gynandra*.

Table S20 Genes that regulate vasculature development, C_4 photosynthesis and heat shock response were mostly derived from WGD in *G. gynandra*.

Table S21 Assembly and annotation of *G. gynandra* genomes in the current and the recent studies.

Table S22 Abbreviations of genes and protein enzymes in this study.

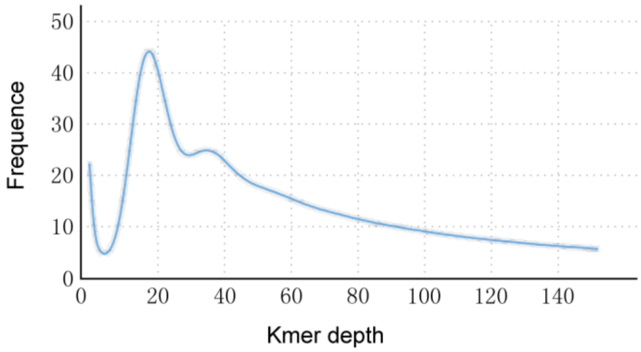

Figure S1 Statistics for the frequencies and depths of 15 *k*-mers in the *G. gynandra* genome. The frequency indicates a bimodal curve caused by heterozygosity. X and Y axes show *k*-mer depth and frequency, respectively. Based on the formula Genome size = *k*-mer number/*k*-mer depth, the genome size of *G. gynandra* was measured to be 997.61 Mb.

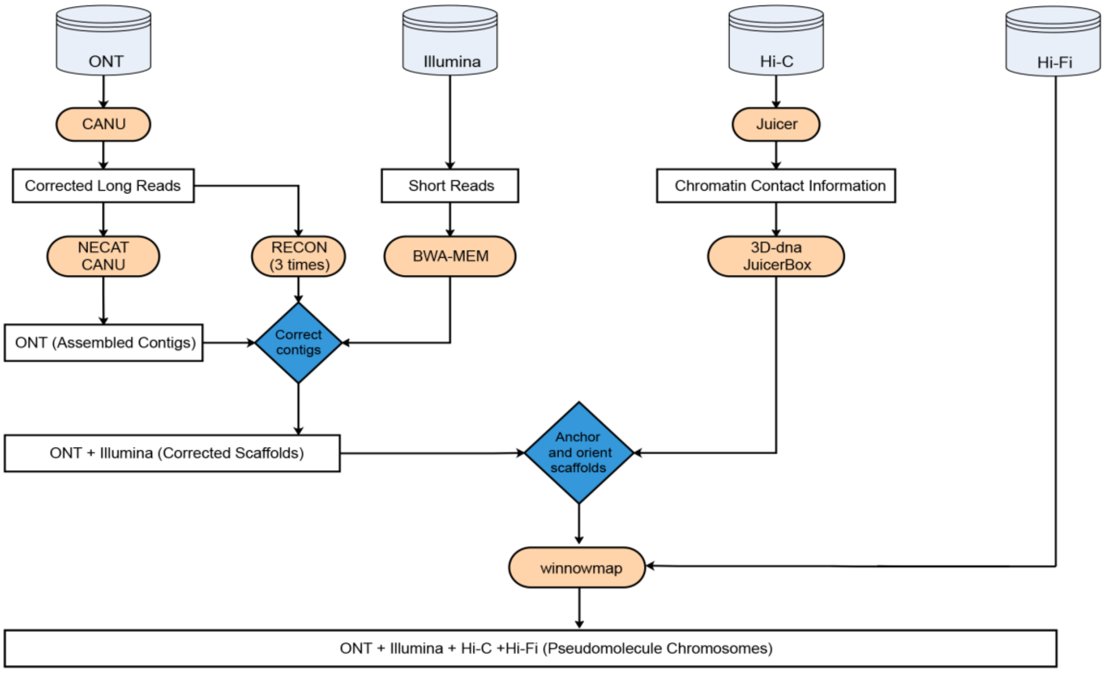

Figure S2 Overview of the processing pipeline for assembling the *G. gynandra* genome. Three different datasets, Oxford Nanopore Technology (ONT) reads, Illumina short-reads and Hi-C mapping, were utilized for hybrid assembly strategy. ONT reads were used to performed contigs assembly. Subsequently, the ONT reads and Illumina short reads were used to correct these scaffolds, and Hi-C mapping was used to anchor and orient the scaffolds into pseudomolecule chromosomes. PacBio HiFi sequencing technology and the corrected HiFi reads along with the corrected ONT reads were used for gap filling, generating the final pseudo-chromosome-length T2T genome assembly for *G. gynandra*.

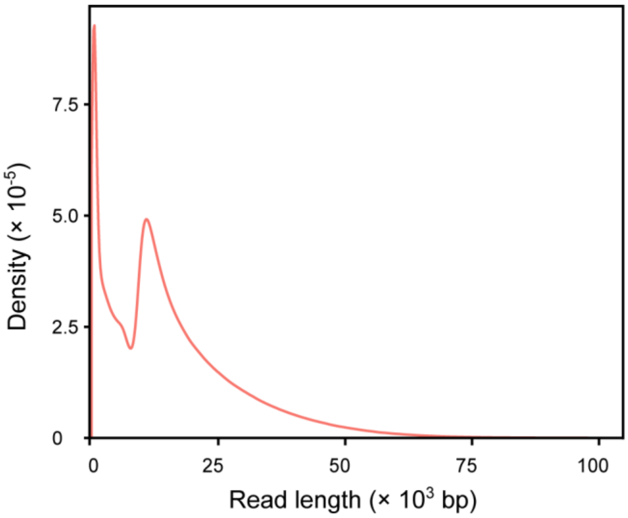

Figure S3 Length distribution of ONT sequencing reads. The X axis and Y axis show length and frequency, respectively.

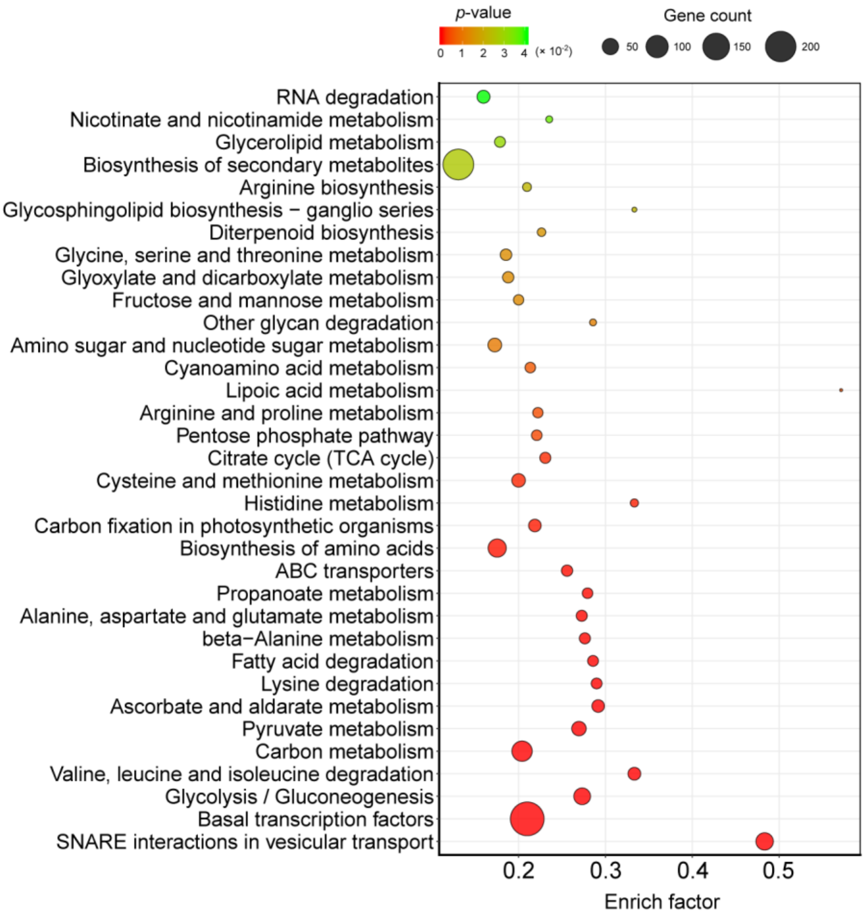

Figure S4 KEGG functional enrichment analysis of gene families specific to *G. gynandra*. The size of the circle indicates enriched gene number of pathway. The color of the circle indicates the enrichment *p*-value of pathway. The significant cutoff for the enrichments was set as *p*-value < 0.05.

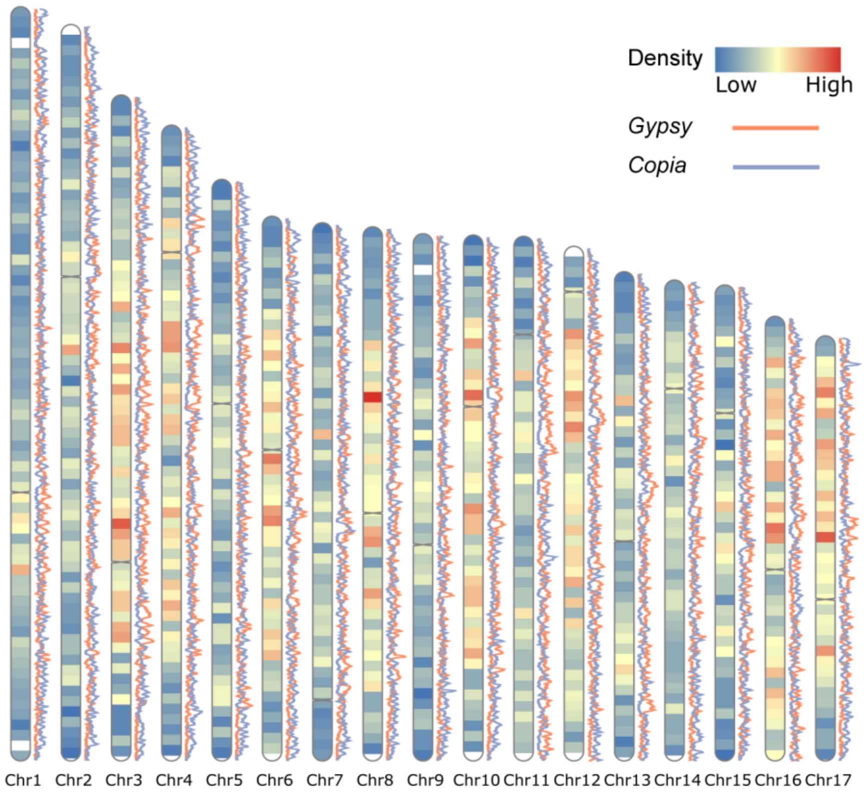

Figure S5 The density distribution of intact LTRs, *Gypsy* and *Copia* in each chromosome of *G. gynandra*. The window size was 100 kb for *Gypsy* and *Copia*, and 1 Mb for intact LTRs. The color from blue to red on the bar density scale indicates a density of LTR-RTs from low to high. These data were visualized using the R package RIdeogram.

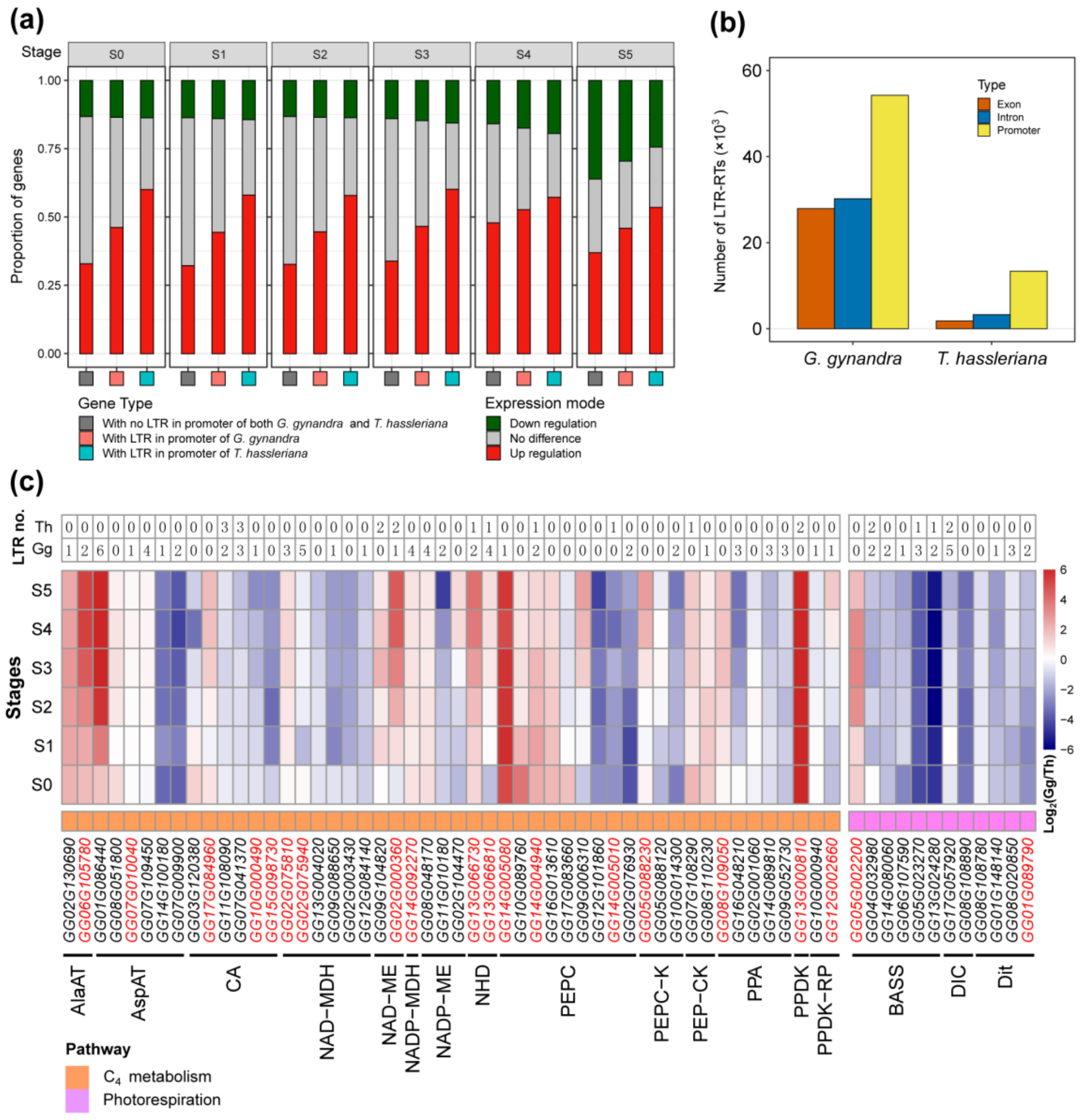

Figure S6 Genomic distribution of LTR-RTs and their impacts on expression patterns of the C$_4$ pathway-associated genes in *G. gynandra* compared to *T. hassleriana*. **(a)** Histograms showing effects of absence or presence of LTR-RT insertions on the gene expression patterns in *G. gynandra* compared to *T. hassleriana*. The 20,094 homologous gene pairs identified between *G. gynandra* and *T. hassleriana* were used for expression analysis. The genes were classed into three groups including genes with no LTR-RT in promoters of both *G. gynandra* and *T. hassleriana* (dark grey), genes with LTR-RT only in promoters of *G. gynandra* (pink), and genes with LTR-RT only in promoters of *T. hassleriana* (blue). The analysis was performed with RNA-seq data of leaves at six developmental stages (from young to mature, S0 to S5) for these two *Cleome* species (Figure 2g). The gene expression levels were normalized with the upper quartile normalization procedure using the youngest S0 leaf stage of *G. gynandra* as the reference. No difference (light grey) is defined if the levels of gene expression show lower than a 1.5 fold change between *G. gynandra* and *T. hassleriana*. The X-axis and Y-axis indicate leaf developmental stages and the proportion of genes in each regulation category, respectively. **(b)** Numbers of LTR-RTs inserted in exon, intron and promoter regions of the *G. gynandra* or *T. hassleriana* genome. The 2 kb region upstream of the transcription start site (TSS) was designated as the promoter region of gene. Much more of LTR-RTs insertion in promoter regions than exons or introns seen both in *G. gynandra* and *T. hassleriana*. **(c)** Expression patterns of the C$_4$ pathway-related genes with altered number of LTR-RTs in *G. gynandra* relative to *T. hassleriana*. The gene names and gene IDs are showed at left and right, respectively. The two columns of numbers show how many LTR-RTs were found in the promoter region of each gene in *G. gynandra* and *T. hassleriana*, respectively. The right panel shows the ID of each gene copy in *G. gynandra*. The heatmap depicts differential expression ratios of the genes between the two species at various leaf developmental stages (S0 to S5). Color reflects fold differences ($\log_2$ ratios) in gene expression. The pathways of C$_4$ circle (orange) and photorespiration (magenta) are indicated beside the heatmap. LTR no., number of LTR-RTs insertion.

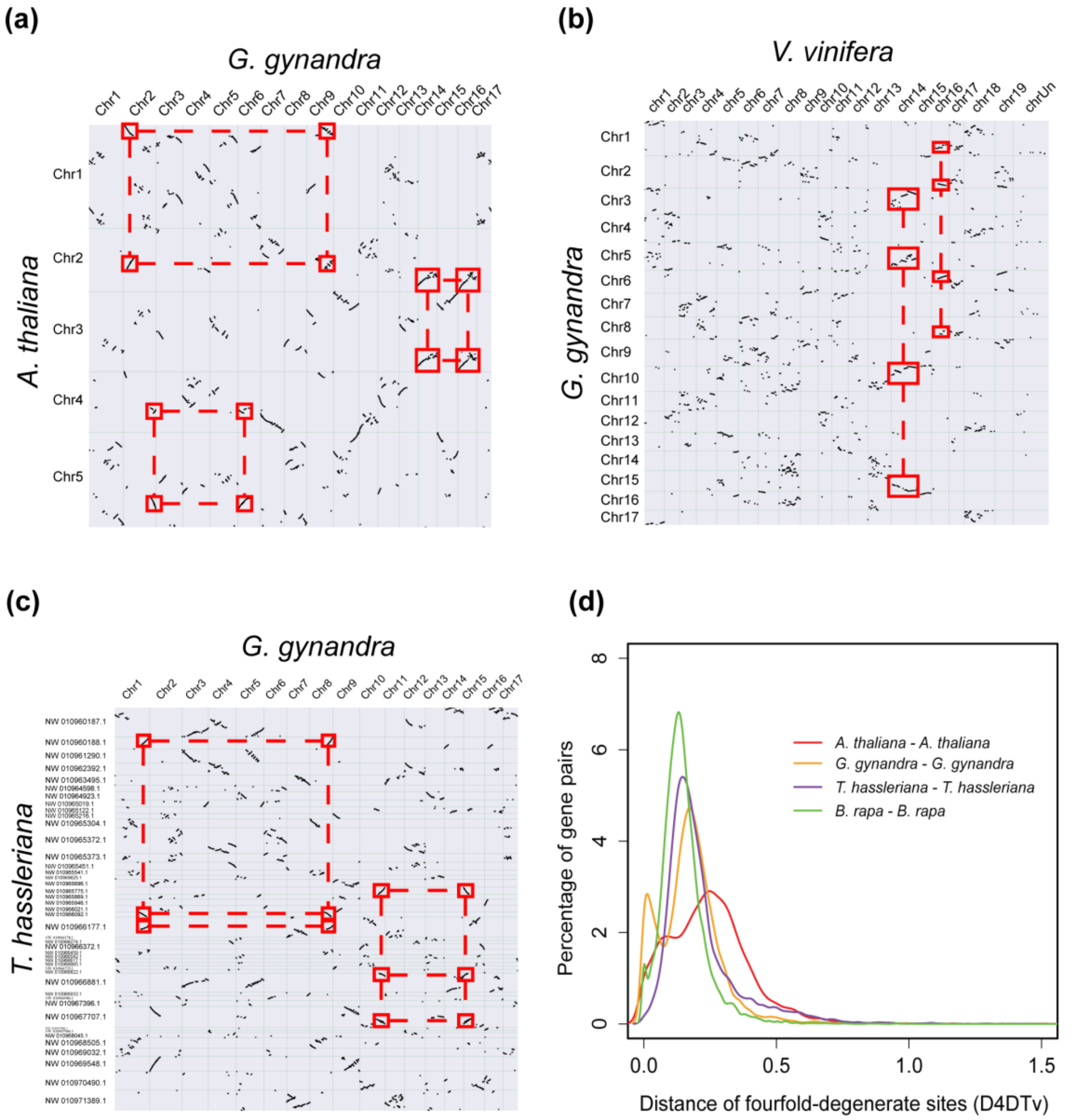

Figure S7 Comparative genomic analysis of the WGD event in *G. gynandra*. **(a)** Syntenic dot plot in *G. gynandra* versus *A. thaliana* genome comparison. The red squares linked by dash lines indicate the synteny blocks between the two genomes. The collinear relationship of 2-to-2 is showed between *G. gynandra* and *A. thaliana*. **(b)** Dotplot figure of the collinear blocks between *V. vinifera* and *G. gynandra* genomes. The red squares linked by dash lines indicate the synteny blocks between the two genomes. The collinear relationship of 4-to-1 is showed between *G. gynandra* and *V. vinifera*. **(c)** Dotplot figure showing the collinear blocks between *G. gynandra* and *T. hassleriana* genomes. Listed are the 45 largest scaffolds of the genome assembly against the 17 *G. gynandra* chromosomes. The red squares linked by dash lines indicate the synteny blocks between the two genomes. The collinear relationship of 3-to-2 is showed between *G. gynandra* and *T. hassleriana*. **(d)** Distribution of 4DTv distance between syntenic orthologous genes within *A. thaliana*, *G. gynandra*, *T. hassleriana* and *B. rapa*. The X axis and Y axis show 4DTv values and the percentage of gene pairs in the syntenic blocks, respectively.

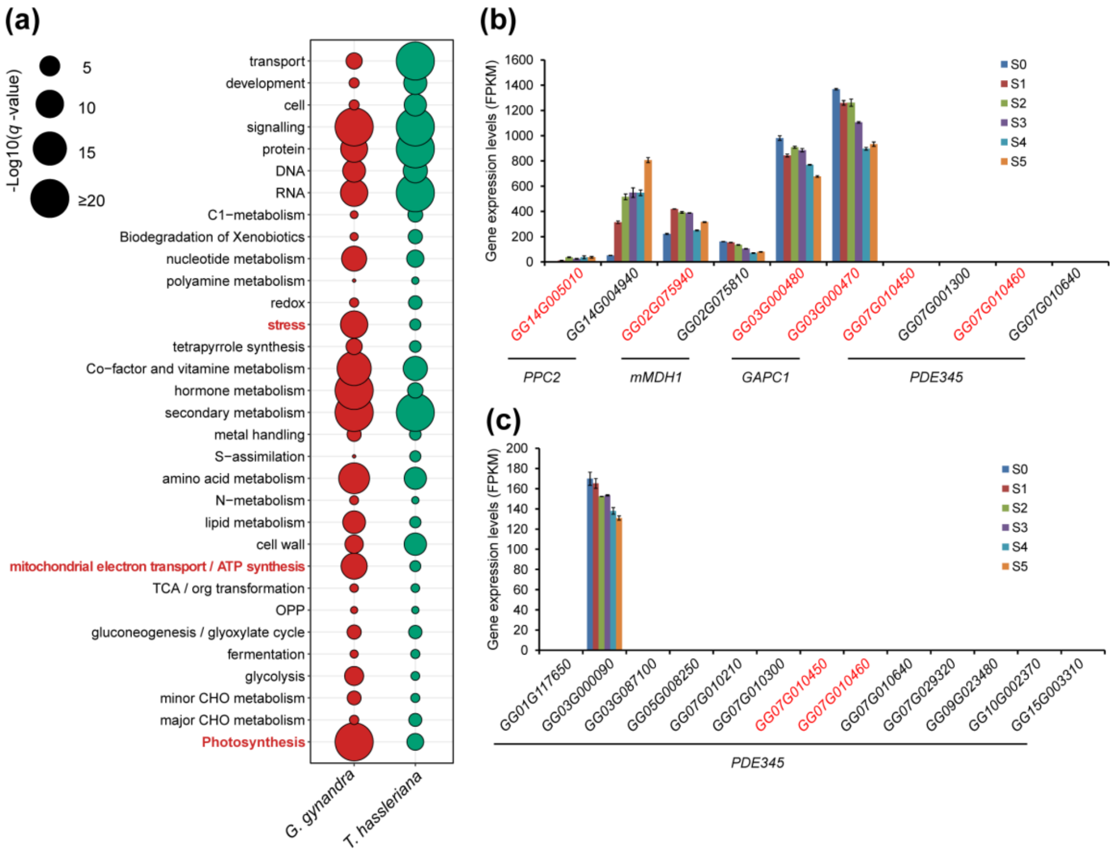

Figure S8 Functional enrichment of WGD-derived genes and expression analysis of TD-derived genes in *G. gynandra*. **(a)** MapMan-Bin enrichment analysis of WGD-derived genes in *G. gynandra* and WGT-derived genes in *T. hassleriana*. The annotation of *G. gynandra* and *T. hassleriana* protein sequences with MapMan terms was performed with the online Mercator (https://www.plabipd.de/portal/mercator4). MapMan4 program was used to conduct MapMan-Bin enrichment analysis for the WGD/WGT-derived genes of the two Cleome species. The size of the circle represents $-\log_{10}(q\text{-value})$ of pathway enrichment. The distinctive MapMan terms in *G. gynandra* as compared to *T. hassleriana* were marked in red bold font. **(b)** Altered gene expression patterns of the carbon fixation pathway in photosynthetic organisms along leaf development gradients for *G. gynandra*. Histograms showing expression levels of the tandem duplicated genes (red) and their paralogues (black). Except for the *PDE345* gene, all the other tandem duplicated genes and their paralogues were differentially expressed in leaves of different developmental stages (from young to mature, S0 to S5). The gene expression analysis was performed as described in Figure 2g. **(c)** Histograms showing expression levels of all the duplicated copies of *PDE345* gene. Note that only one copy of the *PDE345* (*GG03G000090*) was expressed and the tandem duplicated ones (*GG07G010450* and *GG07G010460*) were also not.

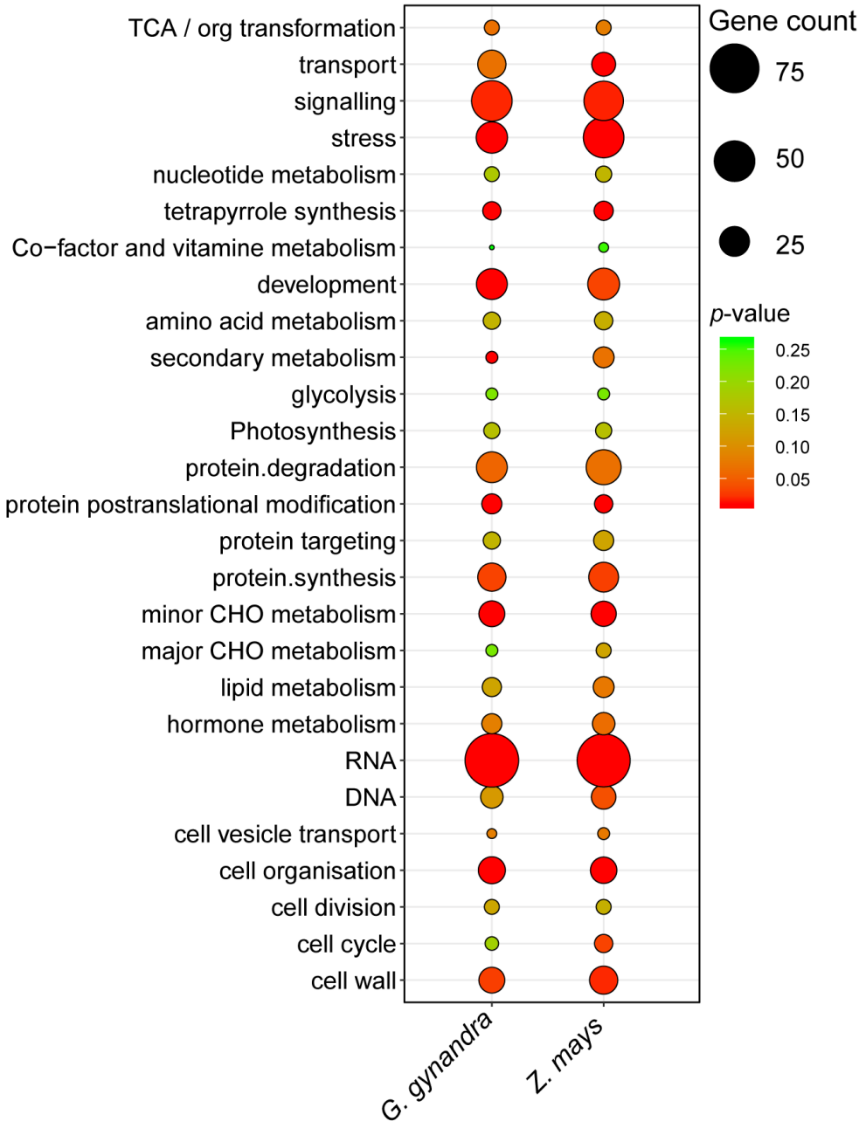

Figure S9 MapMan-Bin enrichment analysis of 221 expanded gene families common to *G. gynandra* and *Z. mays*. The color and size of the circle indicate *p*-value and enriched gene number of pathway, respectively.

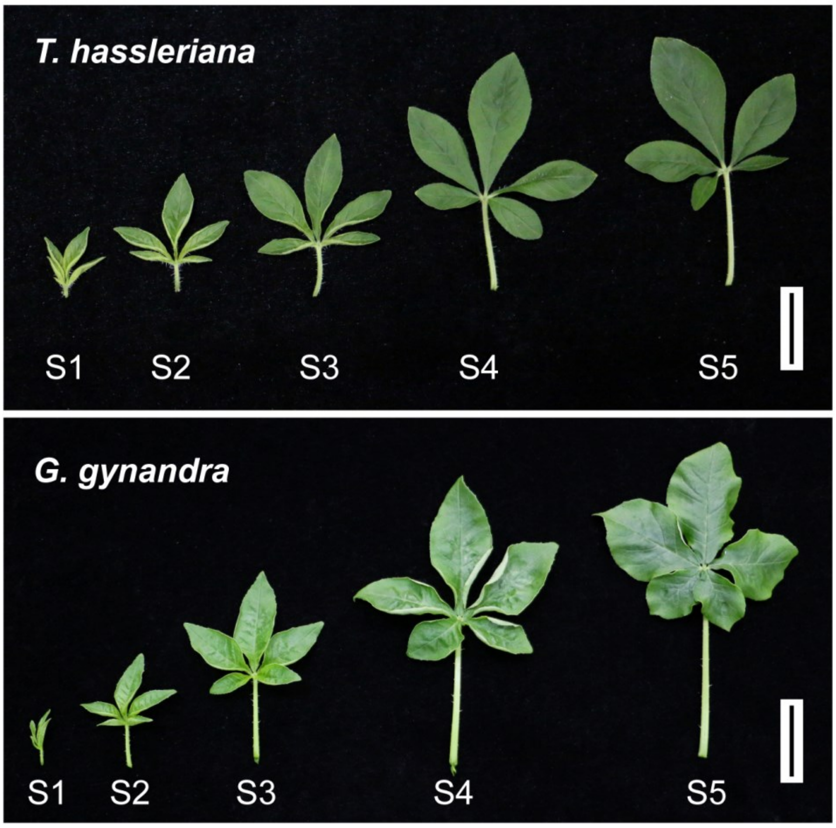

Figure S10 The morphology of leaves at different developmental stages for *G. gynandra* and *T. hassleriana*. Images of leaves from left to right represent stages of 1, 2, 3, 4 and 5 from young to mature for the 5-week-old plants. Bars = 3 cm.

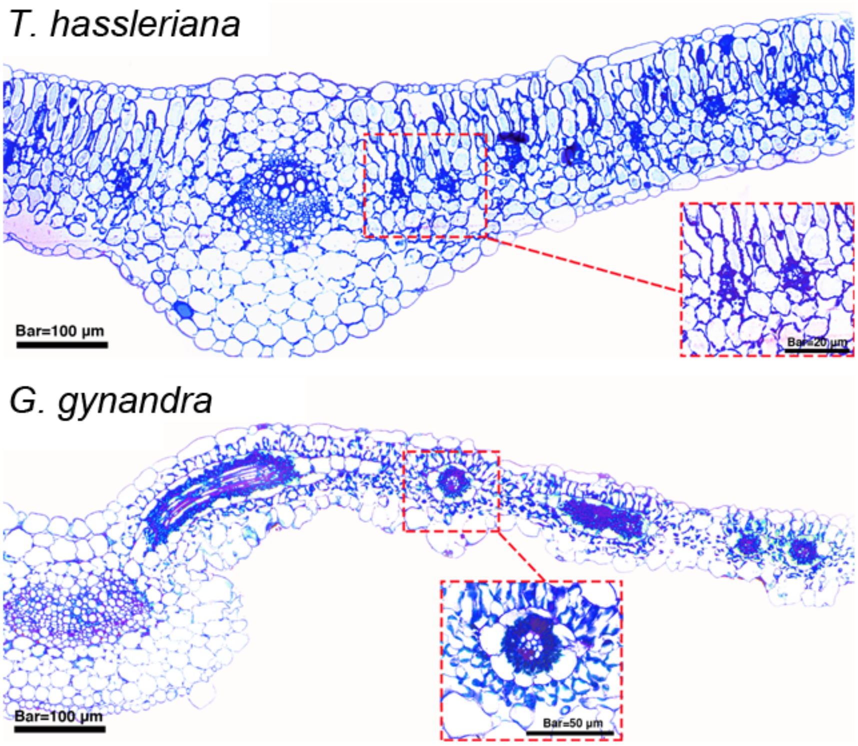

Figure S11 Representative anatomy of the leaf cross-section of *G. gynandra* and *T. hassleriana*. The red dotted boxes indicate the cross-sectional area of bundle sheath (BS) cells.

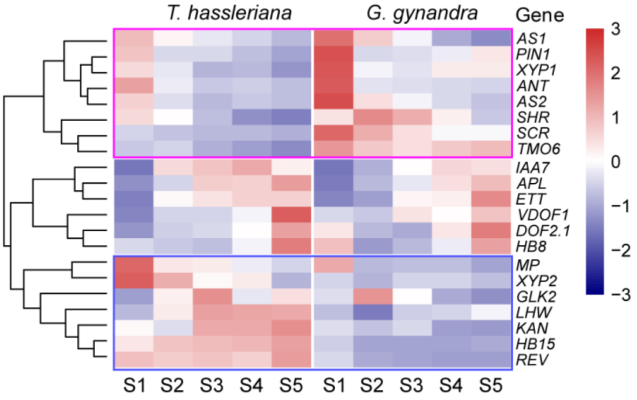

Figure S12 The expression patterns of leaf and vasculature-related regulatory genes along leaf development gradients for *G. gynandra* and *T. hassleriana*. The red box represented the up-regulated genes, while the blue box represented the down-regulated genes in *G. gynandra* as compared to *T. hassleriana* (from young to mature, S1 to S5).

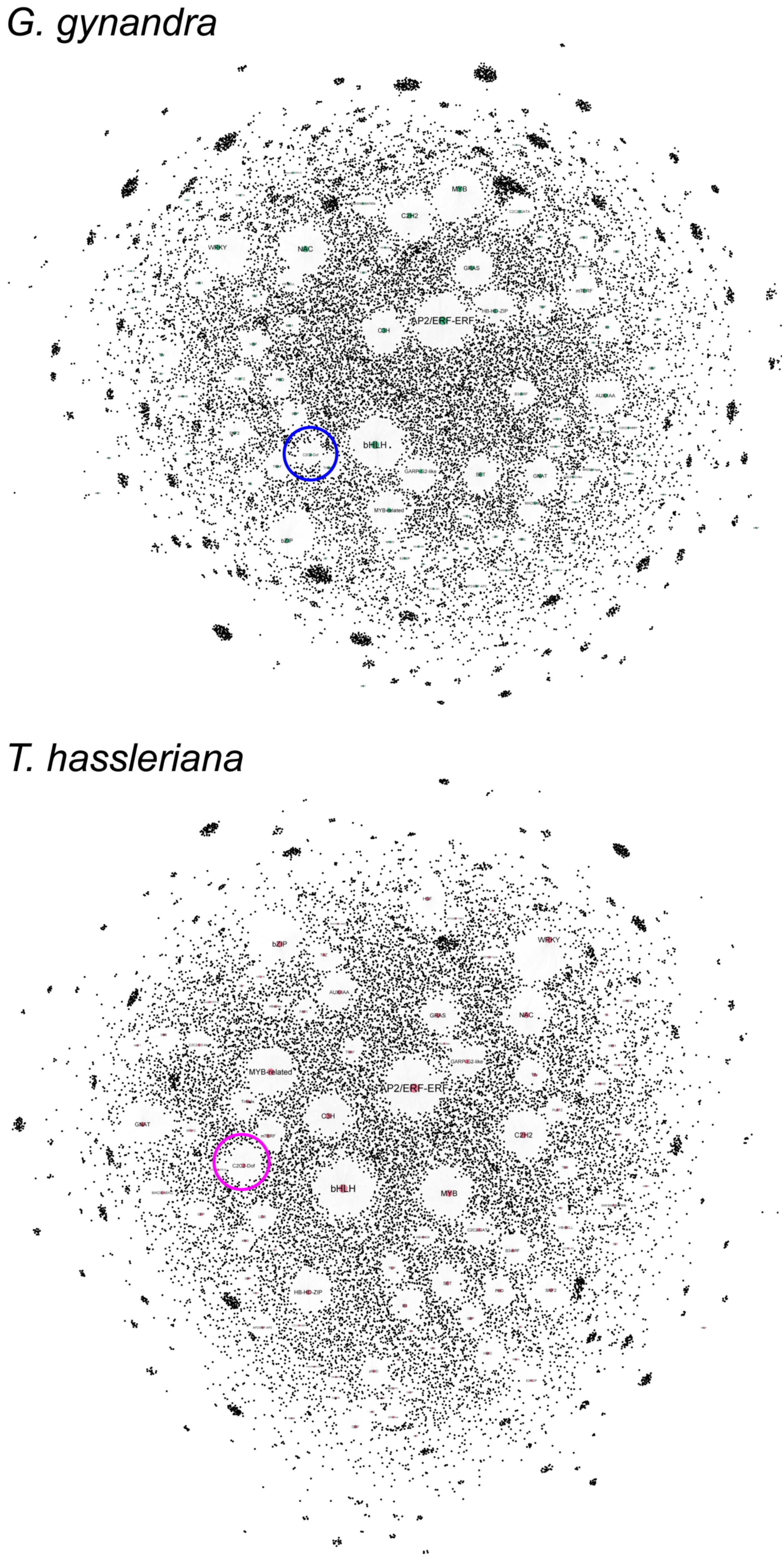

Figure S13 The gene regulatory networks (GRNs) between transcription factors and their candidate target genes in *G. gynandra* or *T. hassleriana*. GRNs were constructed using GENIE3 based on the expression levels of genes during the development of leaves. The networks were visualized with Gephi. The size of the labeled hub genes was proportional to the degree of interconnected nodes. The green and red dots represented the TF families of *G. gynandra* and *T. hassleriana*, respectively. The *Dof* families were highlighted by blue and pink circles in the GRNs of *G. gynandra* and *T. hassleriana*, respectively.

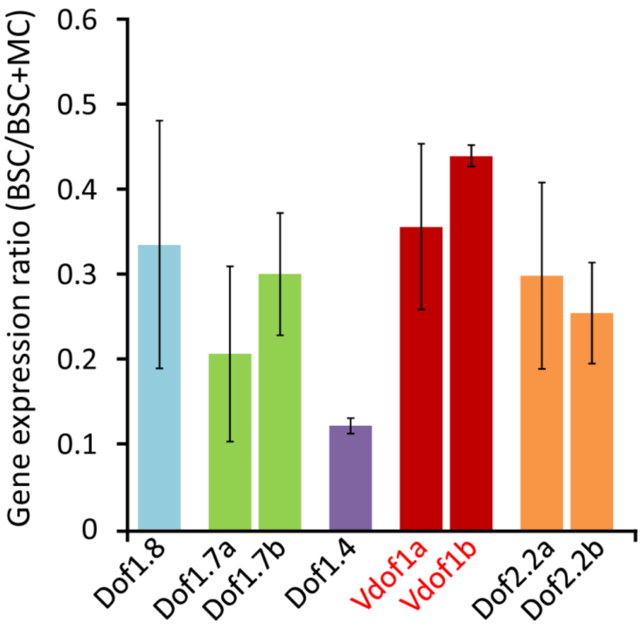

Figure S14 Enrichment of *Vdof1* gene in leaf BS cells. *G. gynandra* has two homologous genes for *Vdof1* (*Vdof1a* and *Vdof1b*; highlighted in red). BSC, bundle sheath (BS) cells; MC, mesophyll (M) cells.

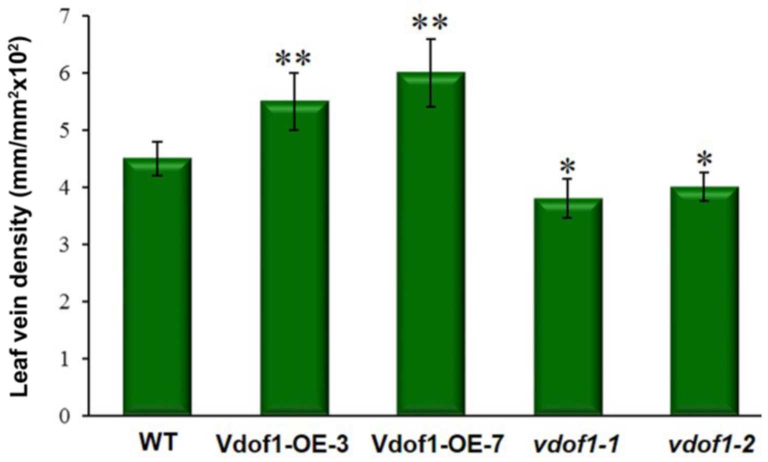

Figure S15 Overexpression of *Vdof1* gene led to enhanced leaf vein density. Analysis of leaf vein density in *Arabidopsis* wild type (WT), *Vdof1* (*AT4G24060*) overexpression lines (OE) and *vdof1* mutants (*vdof1-1*, SALK152104; *vdof1-2*, SAIL554A02).

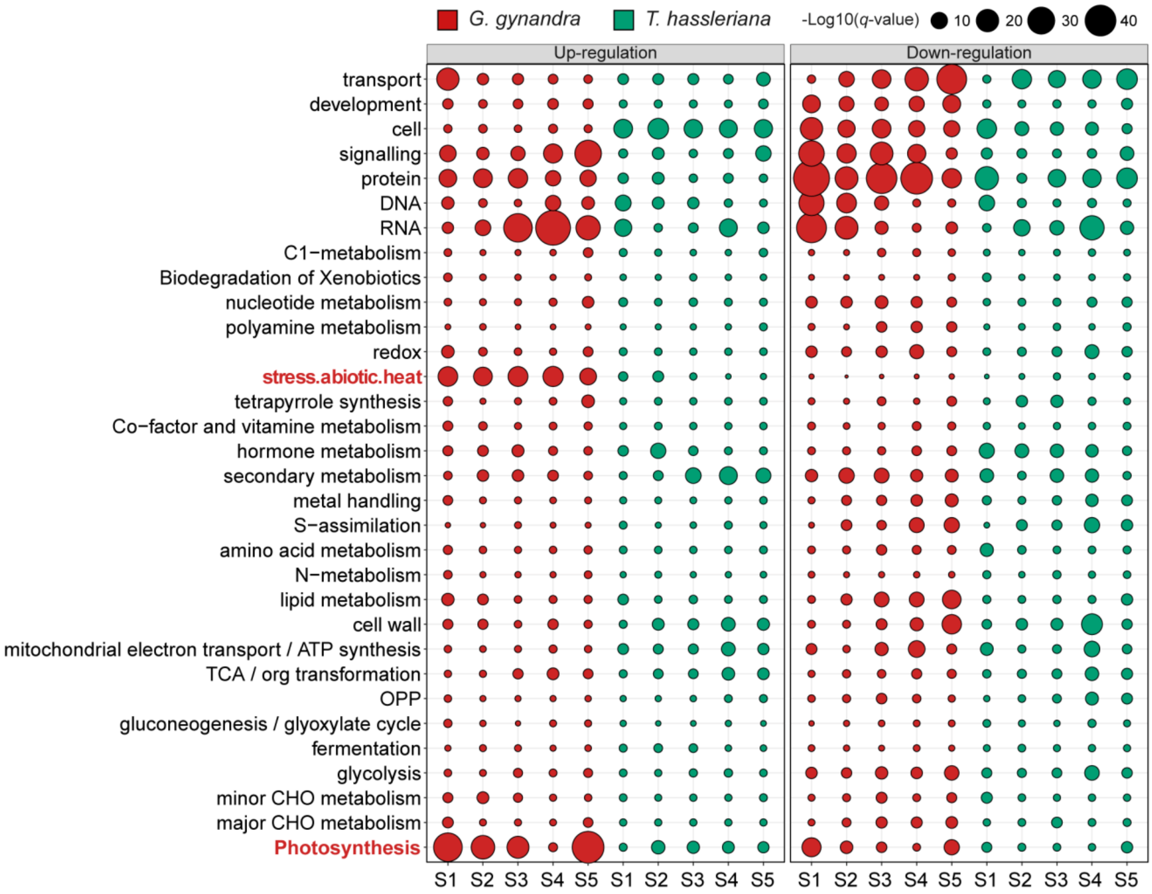

Figure S16 MapMan-Bin enrichment analysis of DEGs at different stages of leaves from *G. gynandra* and *T. hassleriana* subjected to heat stress. The X axis and Y axis indicate leaf developmental stages (from young to mature, S1 to S5) and pathways, respectively. The sizes of the circle indicate -log$_{10}$(q-value) of pathway enrichment. The red and green represent *G. gynandra* and *T. hassleriana*, respectively. The left and right panels show the enrichments of up-regulated and down-regulated genes, respectively. The significantly up-regulated pathways in *G. gynandra* relative to *T. hassleriana* were highlighted by red bold font.

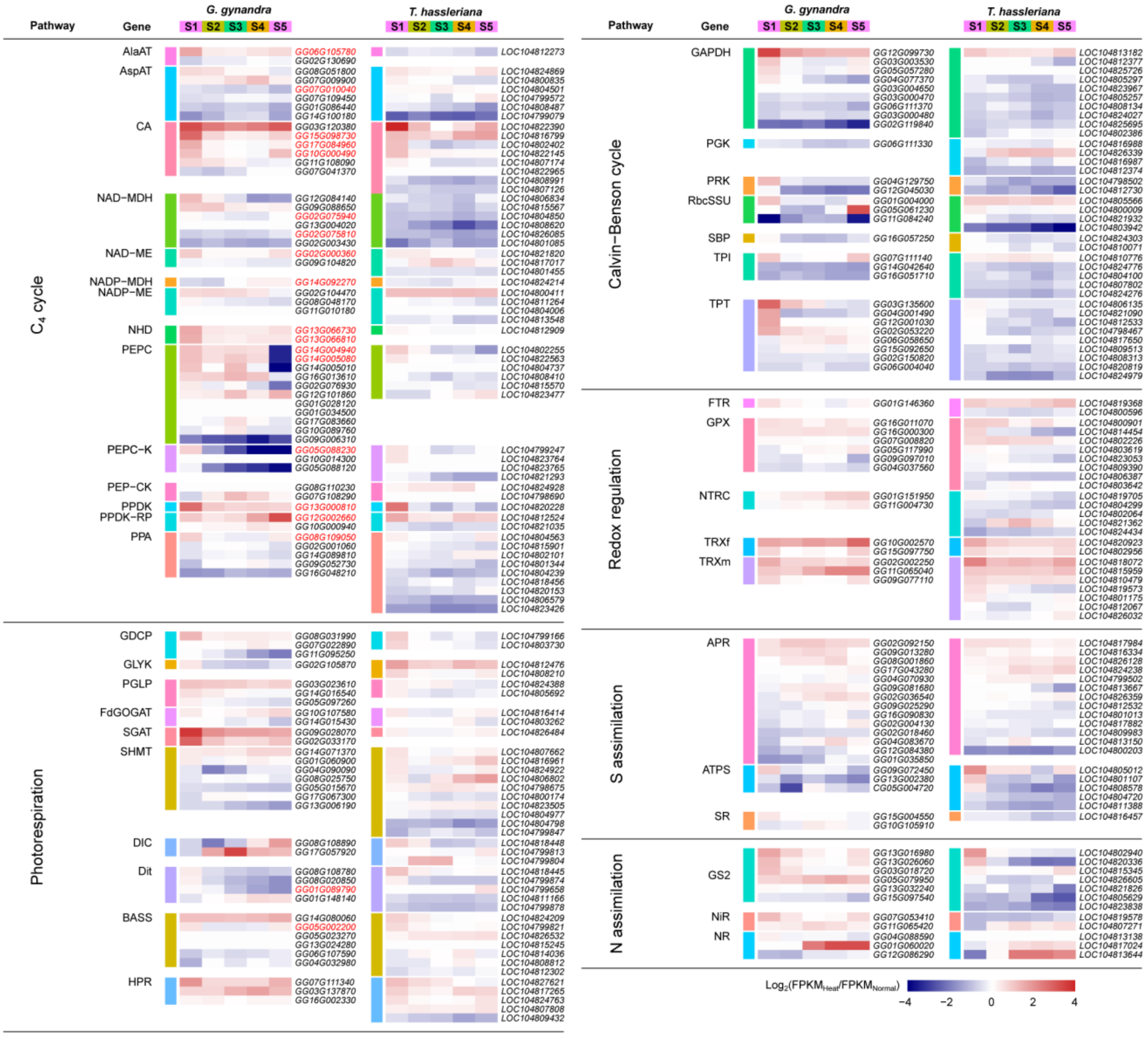

Figure S17 Heatmap showing expression patterns of C4 photosynthesis-related pathway genes under heat stress in *G. gynandra* and *T. hassleriana*. Pathways and member genes are indicated at left. The gene IDs are marked at right. The heatmaps show differential expression ratios of the genes after heat treatments at various leaf developmental stages (from young to mature, S1 to S5) within the two species. The color denotes fold differences (log_2 ratios) in gene expression. FPKM: fragments per kb per million mapped reads.

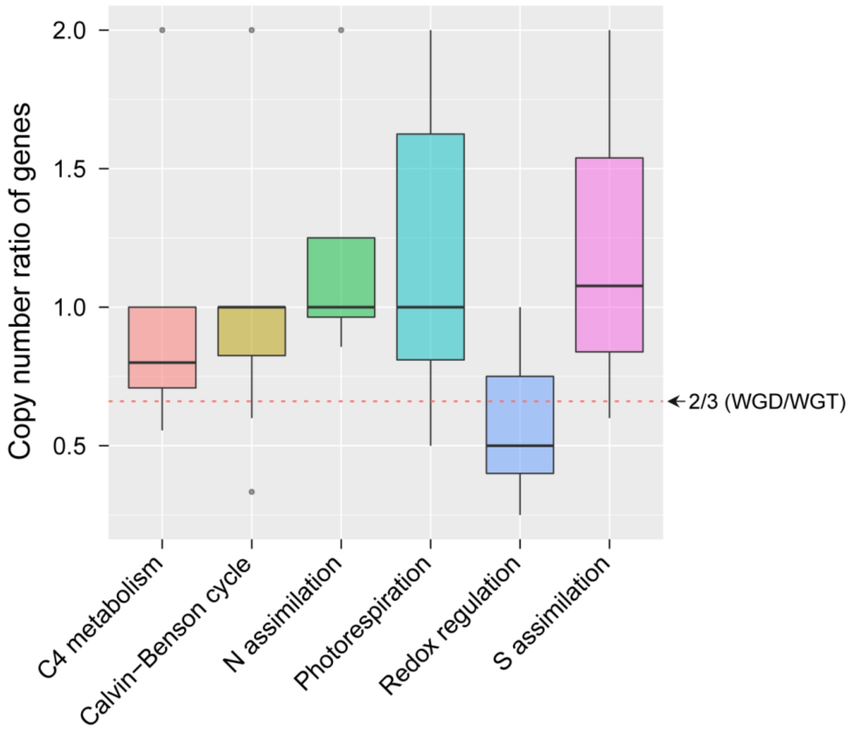

Figure S18 Copy number ratio of genes for C_4 photosynthesis-related pathways in *G. gynandra* as compared to *T. hassleriana*. The red dash line indicates the expected theoretical 2-to-3 relationship for *G. gynandra* (WGD event) versus *T. hassleriana* (WGT event).

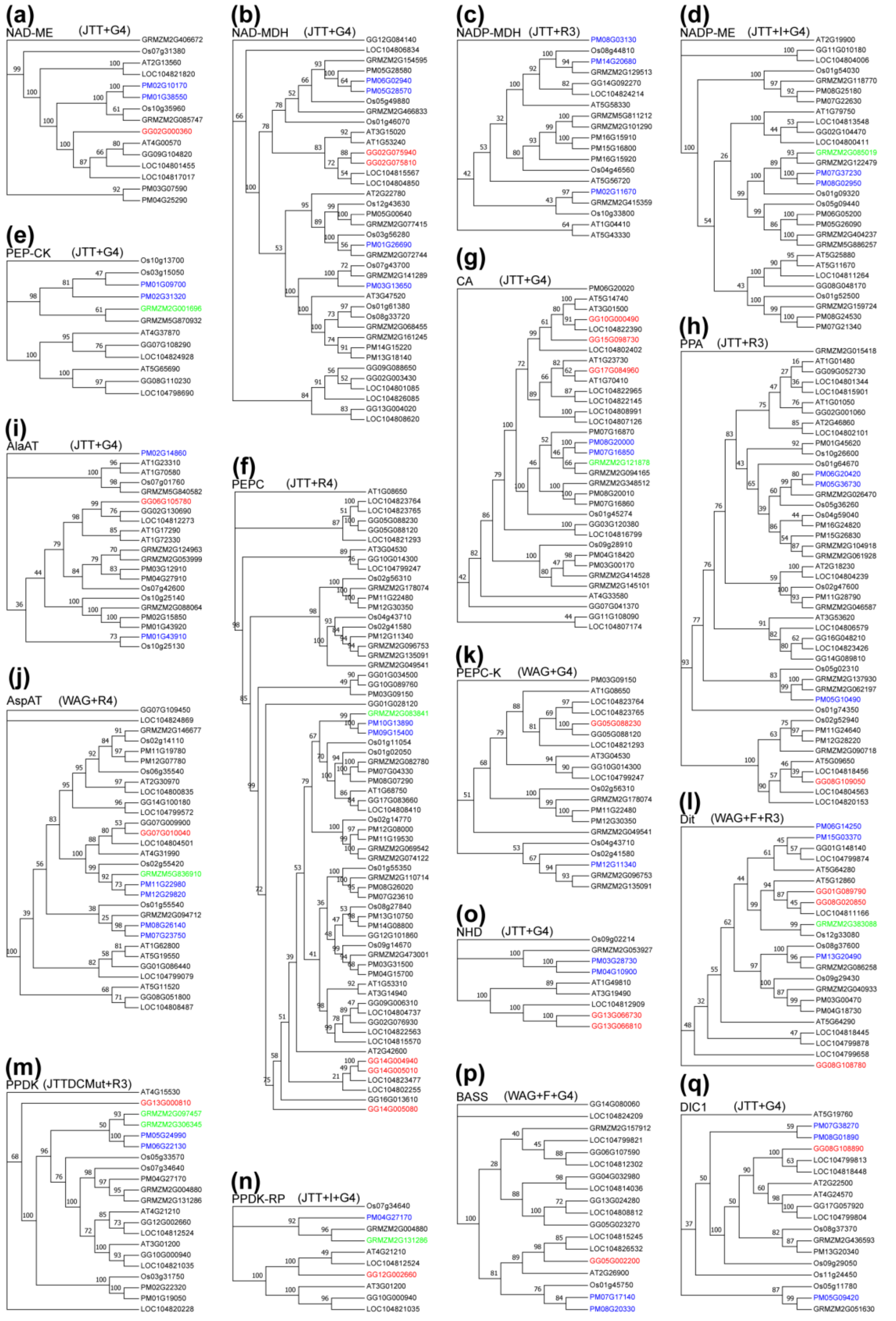

Figure S19 Phylogenetic trees of C_4 photosynthesis-related genes. NAD-ME **(a)**, NAD-MDH **(b)**, NADP-MDH **(c)**, NADP-ME **(d)**, PEP-CK **(e)**, PEPC **(f)**, CA **(g)**, PPA **(h)**, AlaAT **(i)**, AspAT **(j)**, PEPC-K **(k)**, Dit **(l)**, PPDK **(m)**, PPDK-RP **(n)**, NHD **(o)**, BASS **(p)** and DIC1 **(q)**. Maximum-likelihood gene trees were reconstructed from multiple protein sequence alignment using IQ-TREE. Numbers at each branch point indicate the percentage of supports from 1000 bootstraps. The best-fit evolutionary model of each tree is showed in the parentheses beside each gene name. Candidate C_4 genes from *G. gynandra* are in red fonts. Genes from different plant species are distinguished by the leading letters of the accession. Os - rice, PM - *Panicum miliaceum*, GRMZM - maize, GG - *G. gynandra*, LOC - *T. hassleriana*, AT - *A. thaliana*.

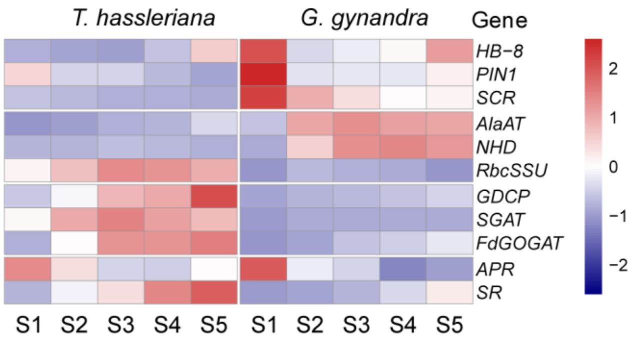

Figure S20 The expression levels of C_4 pathway-related genes with lower copy numbers in *T. hassleriana* than in *G. gynandra* along leaf development gradients. S1 to S5 represented the different developmental stages of leaf from young to mature. The color from blue to red on the color bar scale indicates a normalized gene expression level from low to high.
